# Reversibly Charge-Switching Polyzwitterionic/Polycationic Coatings for Biomedical Applications: Optimizing the Molecular Structure for Improved Stability

**DOI:** 10.1021/acs.langmuir.4c04358

**Published:** 2025-03-05

**Authors:** Sophie H.E. Schneider, Kathrin Lehnert, Marie A. Thome, Annette Kraegeloh, Karen Lienkamp

**Affiliations:** † Chair for Polymer Materials, Department of Materials Science & Engineering, Saarland University, Campus C4 2, Saarbrücken 66123, Germany; ‡ Saarland Center for Energy Materials and Sustainability (Saarene), Saarland University, Campus C4 2, Saarbrücken 66123, Germany; § INM-Leibniz Institute for New Materials, Campus D2 2, Saarbrücken 66123, Germany

## Abstract

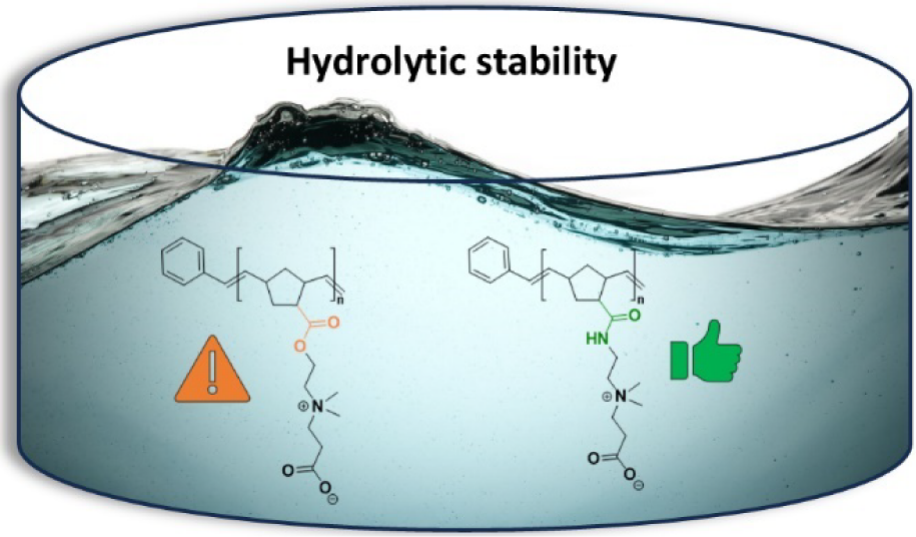

Materials that can be switched between a polycationic/antimicrobial and a polyzwitterionic/protein-repellent state have important applications, e.g., as biofilm-reducing coatings in medical devices. However, the lack of stability under storage and application conditions so far restricts the lifetime and efficiency of such materials. In this work, a polynorbornene-based polycarboxybetaine with an optimized molecular structure for improved hydrolytic stability is presented. The polymer is fully characterized on the molecular level. Surface-attached polymer networks are obtained by spin-coating and UV cross-linking. These coatings are highly uniform and demonstrate charge-switching in zeta-potential studies. Storage stability in the dry state, as well as in aqueous systems at pH 4.5 and 7.4 for 28 days, is demonstrated. At pH 8, hydrolytic degradation is observed. Overall, the materials are substantially more stable than the corresponding ester-based systems.

## Introduction

Durable stimulus-responsive polymeric materials are becoming increasingly interesting for many applications.^1,2^ Devices that are exposed to aqueous environments, from medical applications^3,4^ and water purification to shipbuilding,^5^ often face a limited lifetime due to lack of hydrolytic stability or biofouling. Researchers have worked toward preventing biofouling with so-called stealth materials, most notably oligo(ethylene glycol) (OEG)^4^ and poly(ethylene glycol) (PEG).^6^ These are, to date, the gold standard to prevent the adhesion of proteins, which is the first step in biofilm formation. However, both OEG and PEG are known to be oxidation-sensitive, which limits their areas of use.

Studies show that molecular hydration is a key property to prevent protein adhesion on a material.^7^ Polyzwitterions, which carry an equal amount of cationic and anionic charges on each repeat unit so that they are overall charge neutral, have a higher degree of hydration than even PEG.^4,8^ Thus, polyzwitterions are good candidates for more resilient antibiofilm materials and coatings.^9^ Zwitterionic moieties can be incorporated into polymers in various ways and with different molecular architectures, including main chain and side chain functionalization.^10^ Many studies on the antibiofilm properties of polyzwitterions have been performed on polymers carrying sulfobetaines or carboxybetaines as side chains. These mimic natural compounds such as the zwitterionic lipids of mammalian cells.^11^ Polybetaines are known for their low cytotoxicity; moreover, they efficiently prevent the nonspecific adhesion of proteins on surfaces.^12^

While polyzwitterions are considered chemically inert, polycarboxybetaines are special because their carboxyl group is pH-responsive. This feature can be harnessed for the design of stimulus-responsive coatings whose properties can be switched from polyzwitterionic to polycationic by pH changes.^13−15^ As is well-documented in the literature, many cationic materials are antimicrobial.^16−18^ Thus, surface-attached polycarboxybetaines can be switched from an antimicrobial, cationic state to a protein-repellent, antimicrobial state by protonation.^13,15,19−22^ The field of charge-switching surfaces with antimicrobial and protein-repellent properties has been pioneered by Shaoyi Jiang, initially by using copolymers with carboxylate and quaternary ammonium groups on separate repeat units.^23^ Later studies focused on polycations that were irreversibly hydrolyzed to polycarboxybetaines.^13,24^ The Jiang group then reported a system that could reversibly switch between the polycationic and the polyzwitterionic state—the first system in the literature with these spectacular features.^22,25,26^ Even though the regeneration conditions for the polycations with strong acids were somewhat harsh, which limits potential applications of this system, it is an impressive demonstration of a material being protein-repellent in the polyzwitterionic state and antimicrobial in the polycationic state.

**Figure 1:**
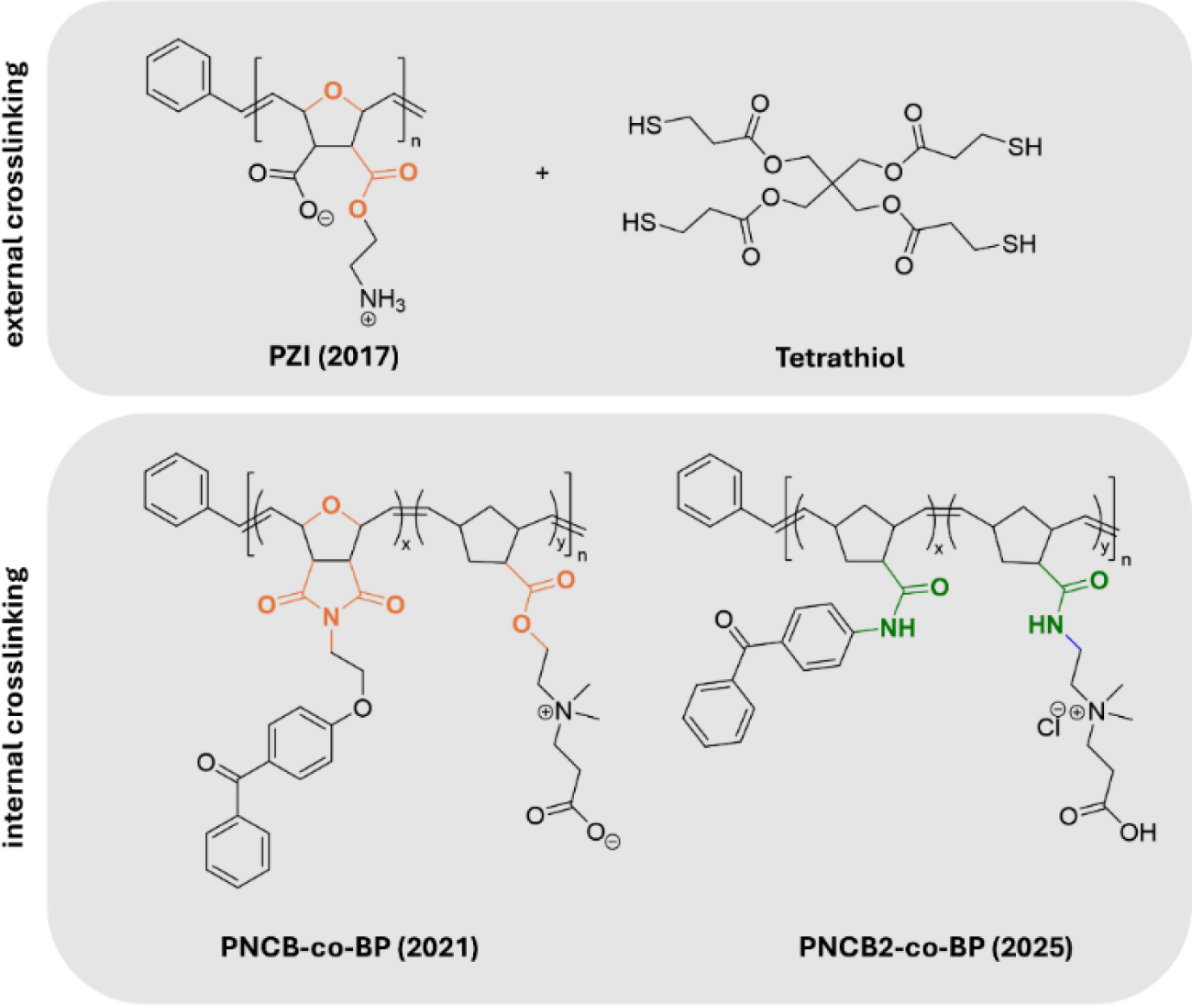
Chemical structures of surface-attached polymer networks with a pH-switchable polycationic, antimicrobial, and a polyzwitterionic, protein-repellent state. **PZI** was cross-linked with the external cross-linker **Tetrathiol** in a thiol–ene reaction; **PNCB-***co***-BP** and **PNCB2-***co***-BP** contained the internal cross-linker benzophenone on a separate repeat unit.

Most poly(carboxybetaines) studied so far have a polymethacrylate backbone. Polynorbornene-based structures are also interesting in this context, as they can carry two side chains per repeat unit, one with an anionic and one with a cationic group. Additionally, it is well-known from the field of polycationic surface coatings that a sound balance between cationic charge and hydrophobicity is needed to make sure that the cationic surfaces are not only antimicrobial but also cell-compatible.^16,27^ This balance can be easily tuned in polynorbornenes, so that on this platform, charge-switchable polyzwitterionic coatings with antimicrobial activity, protein-repellency, and cell-compatibility were obtained.^15,19^ Kurowska et al. presented a poly(oxonorbornene)-based surface-attached polymer network (**PZI**). It was obtained by cross-linking the base polymer, which contained double bonds in the backbone, with pentaerythritol-tetrakis(3-mercaptopropionate) (**Tetrathiol**) in a thiol–ene reaction (Figure 1).^15^

**Scheme 1:**
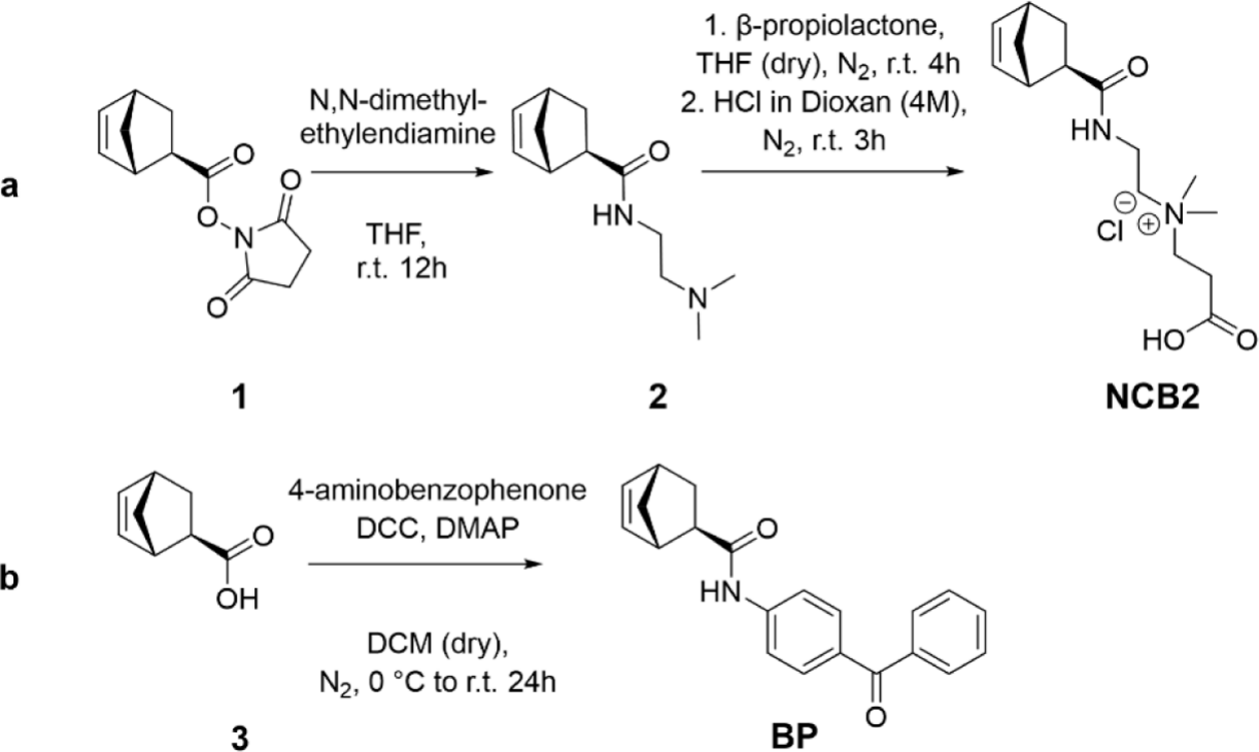
Synthesis of the Target Monomers **NCB2** and **BP**^a^ (a) **NCB2** is obtained in two steps via the active ester **1** and the tertiary amine **2**. The synthesis of **1** is published elsewhere.^32^ (b) **BP** was synthesized using peptide coupling conditions from the carboxylic acid **3**

**PZI** had excellent antimicrobial activity against both *Escherichia coli* and *Staphylococcus aureus* bacteria, which also could not adhere to its surface.^19^**PZI** also prevented the adhesion of lysozyme and fibrinogen. While its resistance to 100% human blood plasma and 10% human blood serum was still very high, it could not prevent the irreversible adhesion of proteins from pure serum.^19^ However, **PZI** was shown to be highly compatible with human keratinocytes.^15^ Unfortunately, **PZI** degraded over time and thereby lost its antimicrobial and protein-repellent features. For this reason, the **PZI** structure was redesigned. In the follow-up system (**PNCB-***co***-BP**, Figure 1), the primary ammonium group, which had led to transamidation reactions within **PZI**, was replaced by a quaternary ammonium group. While **PZI** had the cationic and anionic charges on different side chains, polynorbornene-based **PNCB-***co***-BP** adapted the basic betaine structure, with a cationic quaternary ammonium group and a carboxyl function, separated by an alkyl spacer, on the same side chain.^20^ In this system, the base polymer contained an additional UV-reactive benzophenone group (**BP**, Scheme 1), so that the network formation could be triggered by UV irradiation. This yielded smoother surface-attached polymer networks, which also had a higher gel content than those cross-linked with **Tetrathiol**.^20^ Importantly, with the **PNCB-***co***-BP** system, it could be demonstrated that the protein adhesion in the cationic state was reversible and near-quantitative.^20^ At pH = 3, the protonated, polycationic form of **PNCB-***co***-BP** absorbed a large amount of pepsin, which could be removed by >99% by washing with buffer at pH = 7.4. **PNCB-***co***-BP** was also strongly antimicrobial and nontoxic to human keratinocytes.^20^ Moreover, stability studies showed that the material was stable under storage conditions for >25 weeks, yet degraded after 24 h under physiological conditions and thereby lost its antimicrobial activity. IR spectra revealed that this was due to hydrolysis of the ester group under these conditions.^20^

In this work, we present a redesign of **PNCB-***co***-BP**, with the aim to replace all the remaining labile functional groups in that molecule (marked orange in Figure 1). Instead of using ester groups, the new system **PNCB2-***co***-BP** is based on amide groups (green in Figure 1), which are known for their much higher hydrolytic stability.^21,28^ This should lead to a material for charge-switching polymer coatings with sustained stability under application conditions.

## Experimental Section

### Materials

All materials and chemicals used are listed in the (Supporting Information SI1).

### Instrumentation

Polymer suspensions were centrifuged with a Rotina 380 centrifuge from Hettich (Kirchlengern, Germany) in conical polypropylene Falcon tubes from Greiner Bio-One GmbH (Frickenhausen, Germany). The rotation time was 5 min at 4000 revolutions per minute (rpm). NMR spectra were recorded on a Bruker Avance I 500 MHz spectrometer (Bruker BioSpin GmbH, Rheinstetten, Germany) equipped with a 5 mm TCI Probe (^1^H: 500 MHz, ^13^C: 125 MHz) at 295 K using the standard pulse programs from the TOPSPIN 2.4 software. Chemical shifts (δ) are reported in parts per million (ppm) relative to TMS. CDCl_3_ or D_2_O were used as solvents. Ultrahigh-resolution mass spectrometry (HRMS) was measured via electrospray ionization (ESI) on a Bruker SolariX 7 T MALDI/ESI/APPI FTICR imaging mass spectrometer (Bruker Daltonics GmbH and Co KG, Bremen, Germany). Calibrated size exclusion chromatography (SEC) was performed with 2,2,2-trifluoroethanol (TFE) with 0.05 mol L^–1^ potassium trifluoroacetate (KTFA) as the mobile phase at a flow rate of 1 mL min^–1^ at 30 °C. A PSS column set (FG column for fluorinated organic eluents, Linear-M, 8 × 300 mm, 7 μm) calibrated with poly(methyl methacrylate) standards (PSS, Mainz, Germany) was used as the stationary phase. Infrared (IR) spectra were recorded on a Fourier transform infrared spectrometer (FTIR) with an attenuated total reflection (ATR) mode and a transmission mode (VERTEX 70 V, Bruker Optics GmbH, Ettlingen, Germany). For IR measurements, the polymers were immobilized on double-side polished silicon wafers using a blank wafer as a background. Polymer films were produced by spin-coating on silicon wafers (single-side polished N/Phos <100>, Ø = 100 mm, thickness: 525 ± 25 μm; double-side polished, N/Phos <100> ± 0.5°, Ø = 100 mm, thickness: 600 ± 25 μm, Si-Mat, Kaufering, Germany) or fused silica substrates (20 × 10 × 1 mm, MaTecK, Jülich, Germany) with a SPIN150 spin coater (SPS-Europe, Putten, Netherlands). Networks were cross-linked at 254 nm in Bio-Linker 254 (Vilber Lourmat, Eberhardzell, Germany).

### Physical Characterization

A SE400adv ellipsometer (Sentech Instruments GmbH, Berlin, Germany) was used to measure the thickness of the dry polymer films. The thickness average was calculated from three different measurement spots on the sample. Surface topography was analyzed with the atomic force microscope (AFM) Dimension FastScan from Bruker (Billerica, MA, USA) with commercially available ScanAsyst Air cantilevers (Bruker, Billerica, MA, USA; length 115 μm; width 25 μm; spring constant 0.4 N m^–1^, resonance frequency 70 kHz). All AFM images were recorded in ScanAsyst Air mode. The obtained images were analyzed and processed with the Gwyddion 2.62 freeware software. An electrokinetic analyzer with an integrated titration unit (SurPass, Anton Paar GmbH, Graz, Austria) was used for electrokinetic surface characterization to determine the zeta potential (ζ). Ag/AgCl electrodes were used to detect the streaming current. Before each measurement, the electrolyte hoses were rinsed with Milli-Q water until a conductivity of <0.06 mS m^–1^ was reached. The polymer-coated fused silica samples were inserted into an adjustable gap cell and mounted into the analyzer. An electrolyte solution (1 mmol mL^–1^ KCl) was freshly prepared and adjusted to pH 2.5 with 0.1 g mol^–1^ HCl prior to filling the electrolyte hoses. The gap height of the cell was adjusted to 100–105 μm while rinsing the system for 3 min at 300 mbar before each measurement. Titrations were performed with 0.05 g mol^–1^ NaOH, a target pressure of 400 mbar for the pressure ramp, and a maximum time of 20 s. For each measurement point, four ramps were performed. After titration from acid to basic, the measurement was repeated from basic to acid to assess the stability of the sample. The isoelectric point (IEP) was determined from the curve as the pH value where ζ is 0. The ζ potential at pH 7.4 was defined as ζ_phys_, the ζ potential under physiological conditions. The p*K*_a_ value was calculated as reported previously.^27^

### Stability Studies

Polymer networks were immobilized onto double-sided polished Si wafers. The wafers were stored in air or immersed in different buffer solutions at room temperature. As buffers, citrate (pH 4.5), triethanolamine (pH 7.4), and bicine (pH 8) solutions were freshly prepared. The changes in the polymer films were analyzed by comparing their transmission IR spectra, ellipsometry, AFM, and contact angle measurements.

### Antimicrobial Activity

To test the antimicrobial activity of the polymer networks, modifications of the Japanese Industrial Standard JIS Z 2801 were used as published elsewhere.^29^ Key changes in this assay compared to the original protocol were the reduction of the sample size to 2.5 × 2.5 cm^2^ and a corresponding volume adjustment of the bacterial suspension to 100 μL, containing about 1 × 10^4^ colony-forming units (CFUs) per test or control sample. *Escherichia coli* (DSM498) was used as the test organism. A suspension with approximately 1 × 10^5^ bacteria per mL^–1^ was prepared. 100 μL of the suspension was pipetted onto each of the 2.5 × 2.5 cm*^2^* test samples (polymer-coated silicon wafers) and control samples (uncoated silicon wafers) in three replicates. The samples were covered with polymer foil (2 × 2 cm*^2^*, Hostaphan RNK foil, thickness 50 μm, Mitsubishi Polyester Film GmbH, Wiesbaden, Germany) and incubated for 24 h ± 1 h at 37 °C in a humid chamber. After incubation, the bacteria were detached using 2.5 mL of 0.9% NaCl containing Tween 80 (100 mg L^–1^). After preparation of a dilution series (up to 10^–3^) in 0.9% NaCl plus Tween8 0 (100 mg L^–1^), 3 × 10 μL of each dilution step was spread on tryptic soy agar plates and incubated at 37 °C for 18–24 h. The colony-forming units (CFUs) were counted after the incubation. The log reduction relative to the growth control was reported as *R* = log(*C*) – log(*T*), with *R* = antimicrobial activity, *C* = viable bacteria per cm^2^ on the control sample after 24 h, and *T* = viable bacteria per cm^2^ on the test sample after 24 h.

### Synthesis

The following molecules were synthesized according to literature procedures: 4-(3-triethoxysilyl)propoxy-benzophenone (3-EBP),^30^ [1,3-bis(2,4,6-trimethylphenyl)-2-imidazolidinyliden]-dichloro-(benzylidene)-bis-pyridin-ruthenium(II) (= Grubbs third-generation catalyst, Grubbs^3rd^),^31^**PZI,**^15^ and bicyclo[2.2.1]hept-5-ene-*exo*-2-carboxylic acid *N*-hydroxysuccinimide ester.^32^

#### *N*-[2-(Dimethylamino)ethyl]bicyclo[2.2.1]hept-5-ene-2-carboxamide (**2**)

Bicyclo[2.2.1]hept-5-ene-*exo*-2-carboxylic acid *N*-hydroxysuccinimide ester (16.3 g, 68.4 mmol, 1.00 equiv) and *N,N*-dimethyl ethylenediamine (15.0 mL, 1.39 mmol, 2.00 equiv) were dissolved in tetrahydrofuran (THF) (100 mL) and stirred overnight. The precipitate was filtered off, and the solvent was removed under high vacuum (1 × 10^–3^ mbar). The crude product was redissolved in dichloromethane (DCM) (50 mL) and was washed with water (6 × 25 mL). The layers were separated, and the aqueous layer was extracted with DCM (3 × 50 mL). The combined organic fractions were dried over MgSO_4_, and filtered. The solvent was removed using rotary evaporation and oil pump vacuum. The product was obtained as a colorless solid (12.4 g, 59.5 mmol, 86%). ^1^H NMR (CDCl_3_ with 0.03% v/v TMS, 500 MHz) δ [ppm] = 6.16–6.03 (m, 3H, H1, H1′, H8), 3.36–3.26 (m, 2H, H9), 2.93–2.87 (m, 2H, H2, H5), 2.41 (t, *J* = 6.0 Hz, 2H, H10), 2.23 (s, 6H, H11, H12), 2.01 (ddd, *J* = 8.6, 4.5, 1.6 Hz, 1H, H4), 1.90 (dt, *J* = 11.4, 3.8 Hz, 1H, H3″), 1.76–1.70 (m, 1H, H6″), 1.37–1.26 (m, 2H, H3′, H6’). ^13^C NMR (CDCl_3_, 125 MHz) δ [ppm] = 175.8 (C_quat_, C7), 138.25 (+, CH, C1), 136.17 (+, CH, C1’), 58.03 (−, CH_2_, C10), 47.37 (+, CH, C5), 46.38 (−, CH_2_, C6), 45.28 (+, 2 x CH_3_, C11, C12), 44.64 (+, CH, C4), 41.65 (+, CH, C2), 36.97 (−, CH_2_, C9), 30.56 (−, CH_2_, C3). C_12_H_20_N_2_O M_theo_ 208.1576 g mol^–1^ HRMS (ESI, +) *m*/*z* = 209.1647 [M + H^+^], 210.1687 [M + H^+^ with 1 ^13^C].

#### Bicyclo[2.2.1]hept-5-ene-2-carboxamido-*N*-(2-carboxyethyl)-*N*,*N*-dimethylethan-1-ammonium chloride (**NCB2**)

*N*-[2-(Dimethylamino)ethyl]bicyclo[2.2.1]hept-5-ene-2-carboxamide (9.00 g, 43.2 mmol, 1.00 equiv) was dissolved in THF (anhydrous, 100 mL) under an inert atmosphere. β-Propiolactone (5.52 mL, 90.6 mmol, 2.00 equiv) was added, and the reaction was stirred overnight. The precipitate was filtered off, washed with cooled THF (3 × 20 mL), and dried under high vacuum (1 × 10^–3^ mbar). The dry solid was added to HCl in dioxane (4 mol L^–1^, 130 mL), and the suspension was stirred for 3 h. The solid was filtered off, washed with THF (3 × 20 mL), and dried under high vacuum (1 × 10^–3^ mbar). The final product was obtained as a colorless solid (10.5 g, 33.1 mol, 73%). ^1^H NMR (500 MHz, D_2_O) δ [ppm] = 6.17 (ddd, *J* = 21.1, 5.6, 2.9 Hz, 2H, H1, H1’), 3.68 (t, *J* = 6.5 Hz, 4H, H9, H13), 3.48 (t, *J* = 6.6 Hz, 2H, H10), 3.15 (s, 6H, H11, H12), 3.00–2.88 (m, 4H, H2, H2, H5, H14), 2.17 (ddd, *J* = 9.0, 4.7, 1.5 Hz, 1H, H4), 1.71 (dt, *J* = 11.7, 4.7, 3.5 Hz, 1H, H3″), 1.50–1.28 (m, 3H, H3′, H6). ^13^C NMR (126 MHz, D_2_O) δ [ppm] = 179.98 (C_quat_, C7), 172.95 (C_quat_, C15), 138.41 (+, CH, C1), 136.11 (+, CH, C1‘), 62.15 (−, CH_2_, C10), 59.64 (−, CH_2_, C13), 51.16 (+, 2 x CH_3_, C11, C12), 46.48 (+, CH, C5), 46.00 (−, CH_2_, C6), 43.94 (+, CH, C4), 41.36 (+, CH, C2), 33.35 (−, CH_2_, C9), 30.14 (−, CH_2_, C3), 27.63 (−, CH_2_, C14). C_15_H_25_N_2_O_3_Cl M_theo_ 316.1554 g mol^–1^ HRMS (ESI, +) *m*/*z* = 317.1635 [M + H^+^], 318.1669 [M + H^+^ with 1 ^13^C], 319.1610 [M + H^+^ with 1 ^37^Cl], 320.1642 [M + H^+^ with ^13^C and ^15^N].

#### *N*-(4-Benzoylphenyl)bicyclo[2.2.1]hept-5-ene-2-carboxamide (**BP**)

4-Aminobenzophenone (2.00 g, 10.1 mmol, 1.00 equiv), exo-5-norbornenecarboxylic acid (1.54 g, 11.1 mmol, 1.10 equiv), and 4-dimethylaminopyridine (DMAP) (1.49 g, 12.2 mmol, 1.10 equiv) were dissolved in DCM (anhydrous, 100 mL) under an inert atmosphere. Dicyclohexylcarbodiimide (DCC) (2.53 g, 12.2 mmol, 1.10 equiv) was dissolved in DCM (anhydrous, 10 mL) and added dropwise to the ice-cooled reaction mixture. The reaction mixture was allowed to warm to room temperature and stirred overnight. The precipitate was filtered off, dried in high vacuum (1 × 10^–3^ mbar), and purified by column chromatography (silica, *n*-hexane/ethyl acetate 6/4 to 5/5). The product was obtained as an egg-white solid (2.61 g, 8.23 mmol, 81%). ^1^H NMR (500 MHz, CDCl_3_) δ [ppm] = 7.87–7.72 (m, 5H, H13, H8, H11, H13, H17, H21), 7.71–7.65 (m, 2H, H10, H14), 7.62–7.53 (m, 1H, H19), 7.53–7.43 (m, 2H, H18, H20), 6.18 (dd, *J* = 5.7, 3.0 Hz, 1H, H1’), 6.10 (dd, *J* = 5.6, 3.0 Hz, 1H, H1), 3.07 (dd, *J* = 3.2, 1.6 Hz, 1H, H2), 3.01–2.95 (m, 1H, H5), 2.22 (ddd, *J* = 8.1, 4.3, 1.8 Hz, 1H, H4), 2.04 (dt, *J* = 11.6, 3.9 Hz, 1H, H3″), 1.76 (dt, *J* = 8.2, 1.7 Hz, 1H, H6″), 1.44–1.36 (m, 2H, H3′, H6’). ^13^C NMR (126 MHz, CDCl_3_) δ [ppm] = 195.94 (C_quat_, C15), 174.57 (C_quat_, C7), 142.47 (C_quat_, H12), 138.77 (+, CH, C1), 137.98 (C_quat_, C16), 135.88 (+, CH, C1), 132.77 (C_quat_, C9), 132.36 (+, CH, C19), 131.79 (+, CH, C11, C13), 129.99 (+, CH, C17, C21), 128.41 (+, CH, C18, C20), 118.77 (+, CH, C10, C14), 47.51 (+, CH, C5), 46.40 (−, CH_2_, C6), 46.20 (+, CH, C4), 41.78 (+, CH, C2), 30.76 (−, CH_2_, C3). C_21_H_19_NO_2_, M_theo_ 317.1388 g mol^–1^ HRMS (ESI, +) *m*/*z* = 318.15 [M + H^+^], 319.15 [M + H^+^ with ^13^C], 320.16 [M + H^+^ with ^13^C and ^15^N].

### General Polymerization Procedure

A stock solution of the required reagents was prepared in the degassed solvents. **NCB2** and **BP** were dissolved in a 1:1 mixture of TFE and DCM (10 mL per g monomer) and stirred under an inert atmosphere. The required amount of the stock solutions was mixed in a heat-dried Schlenk tube. The catalyst was dissolved in DCM (3.5 mg mL^–1^) and was added to the monomer solution in one shot. The polymerization was stirred for 30 min, quenched with ethyl vinyl ether (EVE, 1.5 mL), and stirred for 60 min. The solution was directly precipitated in cold diethyl ether, and the polymer was removed by centrifugation. The crude product was redissolved in water and freeze-dried to remove solvent residues. The product was obtained as a colorless or brownish solid.

#### Polymerization of **NCB2**, Yielding Polymer **PNCB2**

**NCB2** (1.00 g, 3.16 mmol, 1.00 equiv), Grubbs^3rd^ (7.10 mg, 0.01 mmol, 0.003 equiv). A 0.56 g portion of the polymer was obtained after precipitation (56%). ^1^H NMR (500 MHz, D_2_O) δ [ppm] = 5.72–5.09 (m, 2H, H1, H1’), 3.82–3.40 (m, 6H, H9, H10, H13), 3.24–2.89 (m, 9H, H2, H11, H12, H14), 2.75–2.45 (m, 2H, H4, H5), 2.08–1.59 (m, 3H, H3″, H6), 1.24 (s, 1H, H3′).

#### Copolymerization of **NCB2** and **BP**, Yielding **PNCB2-***Co***-BP**

**NCB2** (0.86 g, 2.70 mmol, 0.90 equiv), **BP** (0.10 g, 0.30 mmol, 0.10 equiv), Grubbs^3rd^ (6.90 mg, 0.01 mmol, 0.003 equiv). A 0.50 g portion of the polymer was obtained after precipitation (50%). ^1^H NMR (500 MHz, D_2_O) δ (ppm) = 7.97–7.17 (m, 9H, BP), 5.72–5.02 (m, 2H, H1, H1’), 3.90–2.39 (m, 17H, H2, H4, H5, H9 to H14), 2.13–1.58 (m, 3H, H3″, H6), 1.22 (s, 1H, H3′).

The NMR and mass spectra of all monomers and polymers are given in Section SI2 (Figures S1–S20).

### Spin-Coating of Substances

Prefunctionalization: To 5 mL of a solution of 3-EBP in toluene (50 mmol mL^–^,^1^ a solution of 57 mg 3-aminopropyltriethoxysilan (APTES) in 0.5 mL ethanol (EtOH) was added. About 0.2–0.5 mL of the mixture was deposited by spin-casting onto the substrate (1000 rpm, 500 rpm s^–1^, 10 s spinning time). The substrate was then transferred onto a hot plate and allowed to react at 110 °C for 1 h. The substrates were then washed with toluene and EtOH and dried under a N_2_ flow. Polymers: The polymer was dissolved in TFE (30 mg mL^–1^), stirred overnight, and then spin-coated onto the prefunctionalized substrates (3000 rpm, 500 rpm s^–1^, 20 s spinning time). The films were cross-linked for 30 min at 254 nm, extracted in TFE overnight, and dried under a continuous nitrogen flow.

## Results and Discussion

### System Design and Polymer Synthesis

The aim of this work was to design, synthesize, and characterize surface-attached polymer networks that would have not only a switchable bioactivity profile (between a polycationic/antimicrobial and a polyzwitterionic/protein-repellent state) but also sufficient chemical stability. For anticipated use as biofilm-reducing coatings in medical devices, the target materials must be stable under storage conditions (at ambient conditions) and at neutral pH in aqueous surroundings at 37 °C. To that end, polymer **PNCB2-***co***-BP** was designed, in which the proven or assumed labile functional groups of previous charge-switchable systems were eliminated (Figure 1). In that structure, the two charges that constitute the zwitterionic functionality were placed on the same side chain. Instead of connecting the side chain to the backbone via an ester group as in **PNCB-***co***-BP** (Figure 1), the hydrolytically more stable^21,28^ amide bond was chosen. This was expected to increase the resistance against hydrolysis. The synthesis strategy to obtain the norbornene-based monomer **NCB2** is shown in Scheme 1. An activated ester of exo-5-norbornenecarboxylic acid (**1**) was used as the starting material. By reaction of it with *N,N*-dimethyl ethylenediamine in a nucleophilic substitution reaction, precursor **2** with an amide bond and a tertiary amino group was obtained. Notably, only the primary amino group of *N,N*-dimethyl ethylenediamine acted as a nucleophile, while the tertiary amine remained inactive. The thus obtained precursor **2** was quaternized by serving as a nucleophile in the ring-opening of β-propiolactone, yielding the desired zwitterionic monomer **NCB2**. As **NCB2** was to be polymerized by ring-opening metathesis polymerization (ROMP), and the used Grubbs third-generation catalyst does not tolerate carboxylate groups too well,^33^ that monomer was protonated in the last step by suspending it in 4 mol L^–1^ HCl (in dioxane). Dioxane, not being a good solvent for the zwitterionic version of **NCB2**, initially gave an off-white lump, which over time transformed into a suspension when the product was fully protonated.

Previous work had shown that surface-attached polymer networks have fewer defects and are overall smoother if their cross-linker is not an additional reagent (i.e., an external cross-linker like **Tetrathiol** (Figure 1), which could phase separate during processing), but an integral part of the polymer (a so-called internal cross-linker, Figure 1).^20^ For example, benzophenone groups can be used as UV-reactive internal cross-linkers when placed in the polymer side chain. In the previous **PNCB-***co***-BP** system, the benzophenone group was attached to the polymerizable group via a cyclic imide group (Figure 1).^20^ However, the imide can undergo pH-induced ring-opening and is thus unsuitable for the purpose described here.^20^ Therefore, a more stable norbornene-based UV-cross-linkable monomer was designed where the benzophenone unit and the polymerizable group were connected through an amide (Scheme 1). Starting from the carboxylic acid **3**, the benzophenone unit was introduced via a Steglich amination with 4-aminobenzophenone, yielding monomer **BP** in a one-step reaction.

**Scheme 2:**
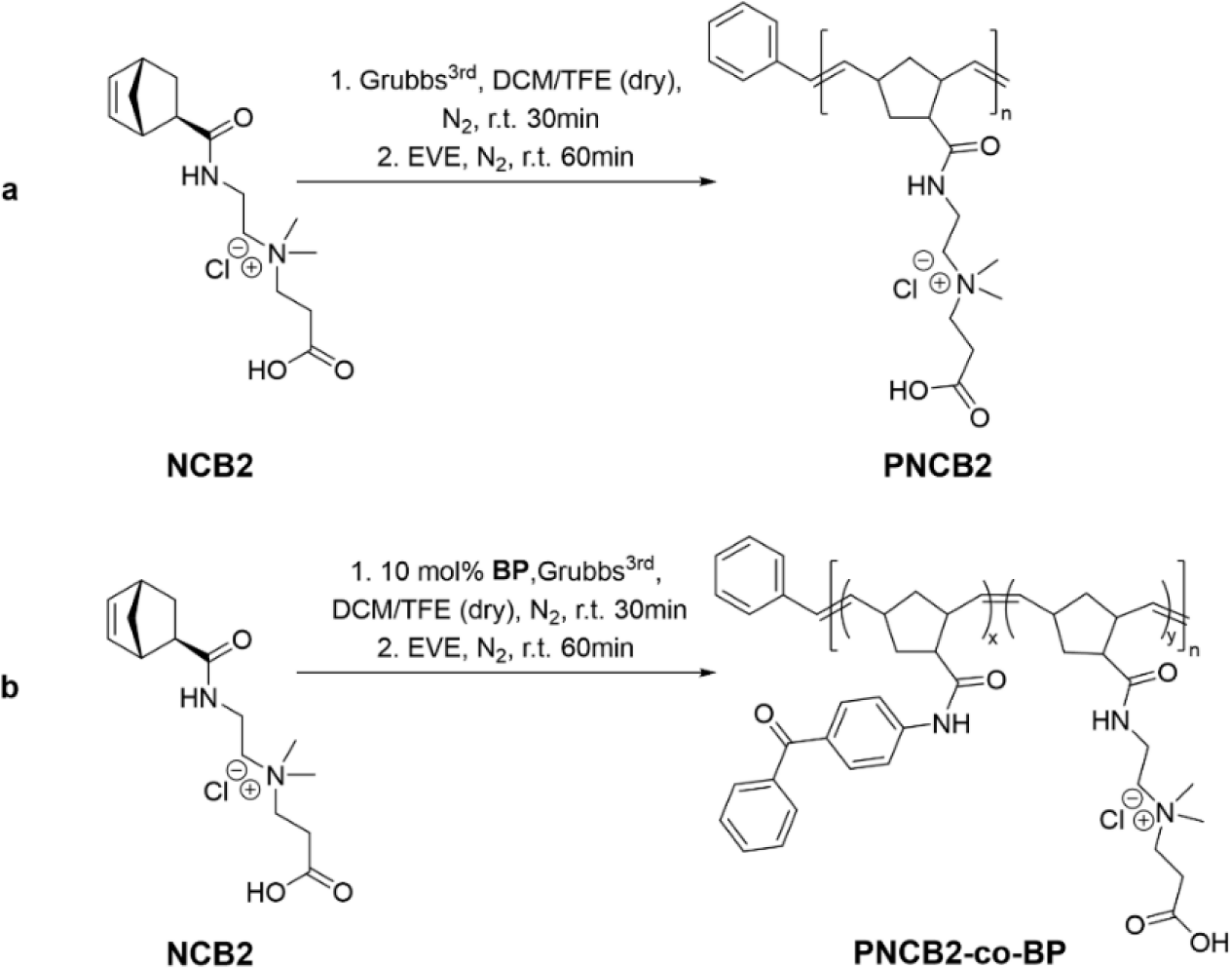
Synthesis of the Target Polymers by Ring-Opening Metathesis Polymerization: (a) Homopolymerization of **NCB2**, Yielding **PNCB2**; (b) Copolymerization of **NCB2** and **BP**, Giving **PNCB2-co-BP**

The target polymers were obtained by ROMP using these monomers. A homopolymer of **NCB2** (= **PNCB2,**Scheme 2a) and a statistical copolymer of **NCB2** and **BP** (= **PNCB2-***co***-BP**, Scheme 2b), each with a molar mass of approximately 100,000 g mol^–1^, were aimed at. In the case of the copolymer, 10 mol % of **BP** and 90 mol % **NCB2** were used. The polymerization medium was a mixture of DCM and TFE. This was a compromise between a solvent that was able to dissolve both the cationic monomer and the polymers (TFE) and a solvent in which the ROMP catalyst was sufficiently stable (DCM). The stability of Grubbs third-generation catalyst in pure TFE is known to be limited to 30 min; therefore, the reaction in the TFE/DCM mixture was also quenched after that timespan by adding ethyl vinyl ether.^34^ The polymers obtained were then precipitated into diethyl ether and recovered by centrifugation, yielding a colorless to brownish solid after drying.

### Molecular Characterization of the Polymers

**Figure 2:**
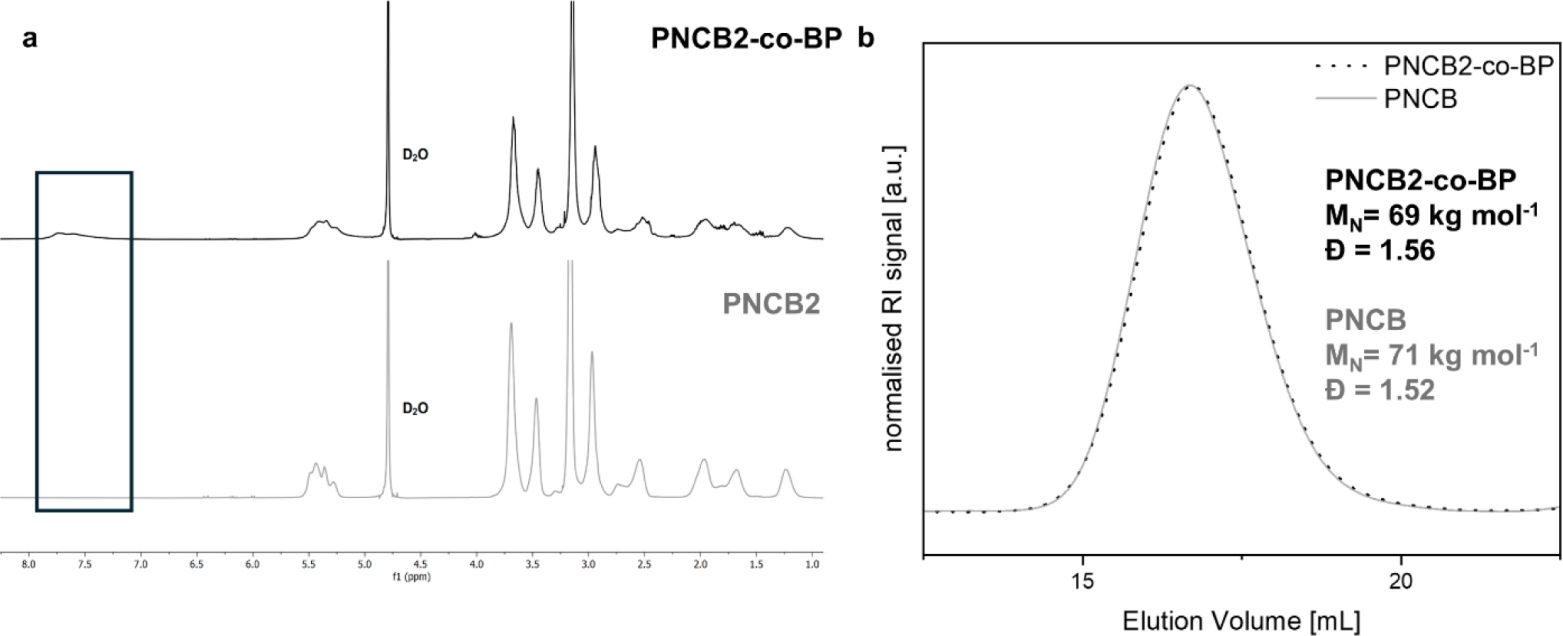
Molecular characterization of **PNCB2-***co***-BP** and **PNCB2**: (a) ^1^H NMR spectra, (b) SEC elugrams were determined by SEC in TFE (with 0.05 mol L.^1^ KTFA, 30 °C, PFG-columns, PMMA standards).

The ^1^H NMR spectra of polymers **PNCB2-***co***-BP** and **PNCB2** are shown in Figure 2a. When comparing these spectra, the most striking difference is that the spectrum of **PNCB2-***co***-BP** contains an extra set of peaks with small intensities between 7.10 and 7.90 ppm. These correspond to the aromatic rings of the benzophenone groups. The other peaks are almost identical to those of the spectrum obtained for **PNCB2**. Thus, the functional groups of both the zwitterionic and aromatic repeat units are present in the spectrum of **PNCB2-***co***-BP**, which is a first indication that the targeted copolymer was indeed obtained. By integrating the signal intensity of the aromatic peaks of the benzophenone units and comparing that data to the total intensity of the polymer double bond peaks from the backbone (5.00–5.60 ppm), the approximate molar ratio of the repeat units was obtained. This calculation gave 9 mol % of **BP** repeat units, which is very close to the targeted 10 mol %. SEC gave elugrams (Figure 2b) with a single, relatively symmetric peak for both the homo- and copolymerned, indicating a monomodal molar mass distribution and reasonably controlled polymerization conditions. This is another indication that **PNCB2-***co***-BP** is indeed a copolymer, or else a second peak (**BP** homopolymer) would be expected in the elugram. The SEC data of **PNCB2-***co***-BP** and **PNCB2**, which were synthesized exactly in parallel, were very similar. The number-average molar masses *M*_n_ obtained were 68,800 and 71,000 g mol^–1^, respectively, and the dispersities (*Đ*) were 1.56 and 1.52. The *Đ* values are broader than would be expected for ROMP, which is due to the previously mentioned stability issue of the polymerization catalyst in the solvent mixture used. Consequently, the molecular masses obtained were also slightly lower than the targeted ones. Nevertheless, the polymer chain lengths were sufficient for the fabrication of polymer networks by cross-linking.

**Figure 3:**
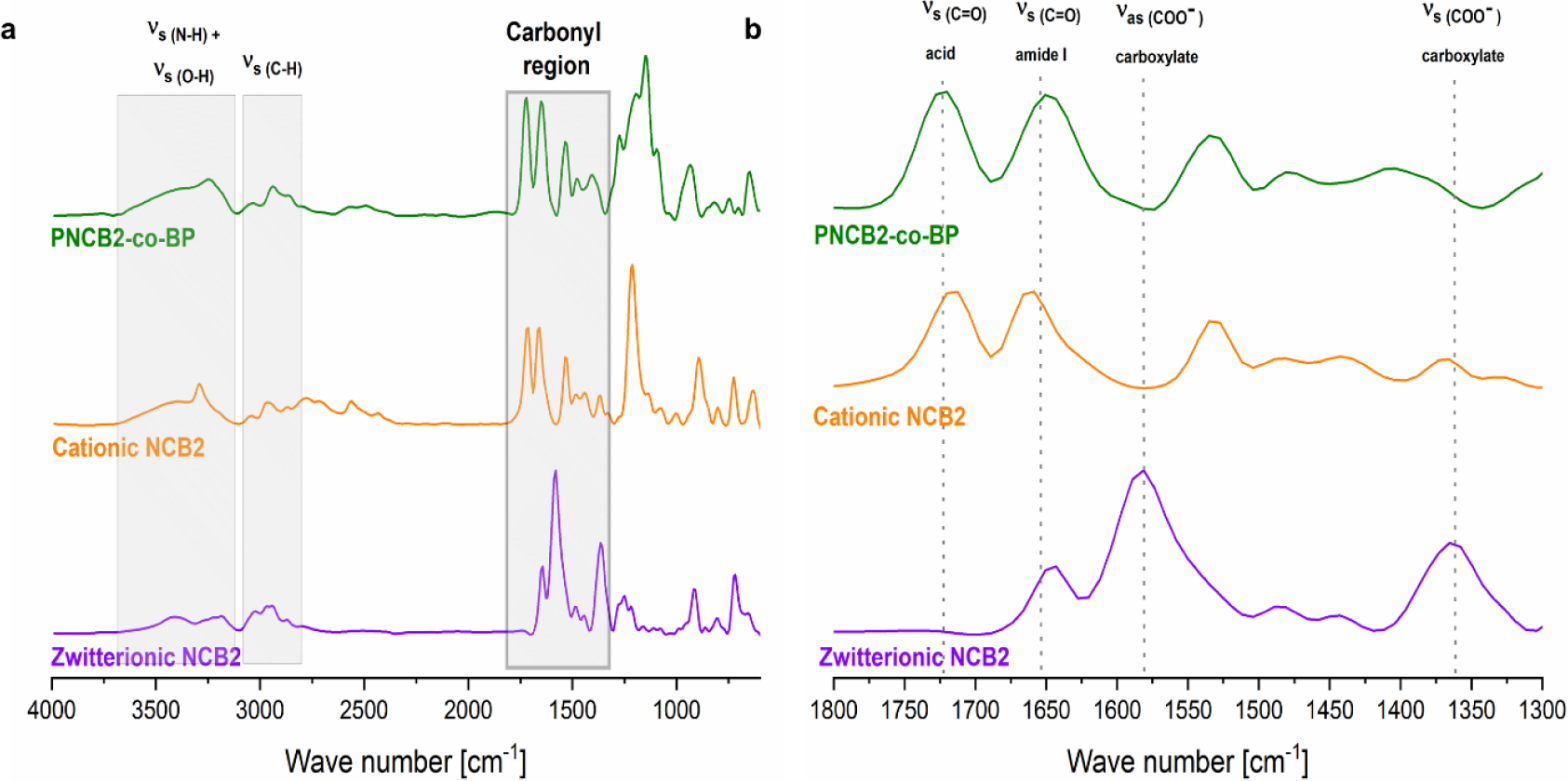
ATR IR spectra of the monomer **NCB2** in its zwitterionic (purple) and cationic state (orange), respectively, and **PNCB-***co***-BP** (green). (a) Full spectra, (b) zoom in on the carbonyl region. The most important regions and peaks are highlighted.

ATR-IR spectra of copolymer **PNCB2-***co***-BP** and monomer **NCB2** in its cationic and zwitterionic forms are compared in Figure 3.

The spectrum of the zwitterionic monomer (Figure 3, purple) contained peaks that were due to the symmetric stretching vibration of ν_s(C=O)_ carboxylate at 1365 cm^–1^, and the corresponding asymmetric stretching vibration of ν_as(C=O)_ at 1581 cm^–1^, respectively. Those bands disappeared in the protonated, cationic state and are replaced by a signal at 1712 cm^–1^ originating from the ν_s(C=O)_ of protonated carboxylic acid (Figure 3, orange). The latter can also be seen in **PNCB-***co***-BP** (Figure 3, green), indicating that the polymer remains protonated during polymerization and after isolation. All three spectra contained a peak of the ν_s(C=O)_ of the amide (amide I) with a maximum between 1660 and 1640 cm^–1^, depending on the exact chemical surroundings.^35^

### Surface Coating and Characterization

**Figure 4:**
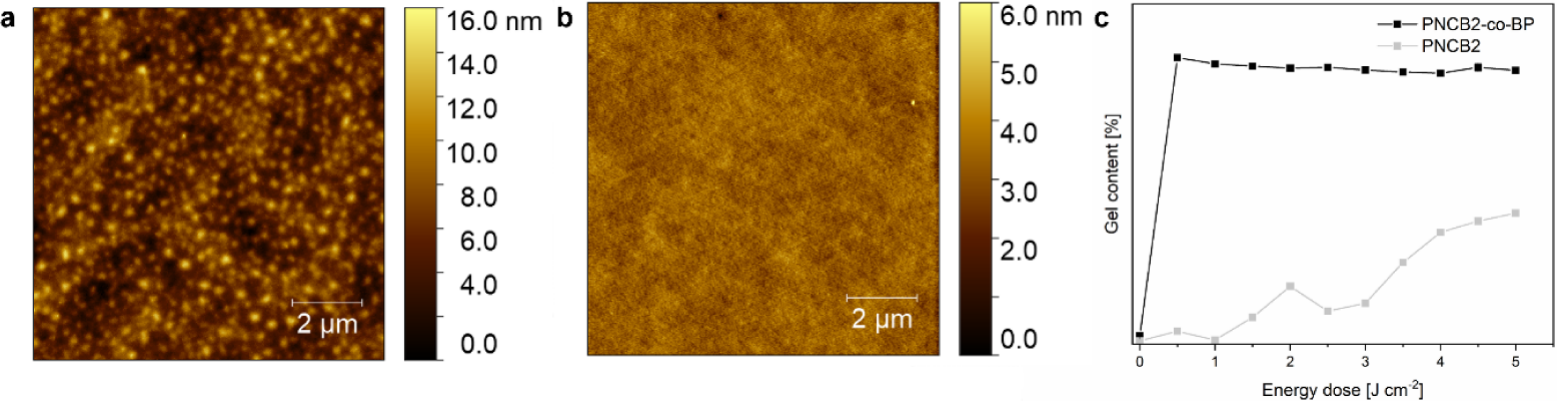
Surface morphology of (a) **PNCB2** and (b) **PNCB2-***co***-BP** imaged by atomic force microscopy (height images). The gel content of both networks is shown in (c).

The targeted surface-attached polymer networks were obtained by dissolving polymers **PNCB2** and **PNCB2-***co***-BP** in TFE and spin-coating the solution onto prefunctionalized silicon wafers or quartz pieces (for the ζ potential measurements). The prefunctionalization procedure of these substrates with a mixture of surface-attached benzophenone groups and amine groups (to increase the surface hydrophilicity) has been described in detail elsewhere.^29^ In the case of homopolymer **PNCB2**, additional UV cross-linker **Tetrathiol** (Figure 1) was added to the spin-coating solution as an external cross-linker. The copolymer **PNCB2-***co***-BP** could be used directly due to its internal UV-active benzophenone groups. The environmental conditions during spin-coating had to be carefully controlled, as both too high temperature and humidity caused dewetting of the solution from the surface during processing. After being spin-coated at 3000 rpm, the obtained polymer films were directly irradiated with UV light at a wavelength of 254 nm. By this activation, a surface-attached polymeric network was formed. In the case of **PNCB2**, a thiol–ene reaction^36^ between the thiol groups and the double bonds of the polymer took place, as reported previously for other polymers.^15,19,20^ In the **PNCB2-***co***-BP** copolymer, a C,H insertion reaction (CHic)^37^ between the benzophenone groups and aliphatic C–H bonds of the polymer backbone took place.^37^ For both polymers, surface attachment was achieved by CHic reactions between surface-attached benzophenone groups and the C–H bonds of the polymer backbone. The cross-linking efficiency of each system was quantified by calculating the gel contents of the networks using a previously reported procedure.^38^ In short, the coatings were irradiated with different energy doses and then extracted with a good solvent (TFE) to remove any chains that were not covalently attached. The coating thickness was measured by ellipsometry before and after extraction. The ratio between the two thickness values multiplied by a hundred yields the gel content. The results are shown in Figure 4. **PNCB2** had a maximum gel content of 42% after irradiation with an energy dose of 5 J cm^–2^. In contrast, **PNCB2-***co***-BP** with the internal benzophenone cross-linker had a gel content of 90% already at an energy dose as low as 0.5 J cm^–2^. These results are in good agreement with the data obtained for the previously reported **PNCB** system and its copolymers.^20^ Notably, the gel content of **PNCB2-***co***-BP** exceeds that of **PNCB-***co***-BP** by 22–27%. This could be due to the optimized structure of the polynorbornene-based benzophenone repeat unit used in this study, which contains fewer heteroatoms than the previously used oxanorbornene-based cross-linker repeat unit. As shown recently, the gel content of poly(oxanorbornenes) with many additional heteroatoms in the side chain is limited because the carbon–hydrogen bonds directly next to heteroatoms are unreactive.^38^ Due to its higher gel content, the overall layer thickness of **PNCB2-***co***-BP** was also higher than that of **PNCB2,** which was cross-linked with the **Tetrathiol**. For a single coating application, **PNCB2** layers had a thickness of 108 nm, while those of the copolymer reached 273 nm (a factor of 2.5 higher).

The surface topography of the networks was analyzed by AFM (Figure 4, height images taken in the ScanAssist mode). Quadrature and Inphase images^39^ can be found in Section SI3, Figures S21–S24). Both the **PNCB2-***co***-BP** and the **PNCB2** surfaces were smooth, with rms roughnesses of 1.5 ± 0.1 and 0.4 ± 0.0 nm, respectively. However, further studies were conducted with **PNCB2-***co***-BP** only, as it gave an overall higher layer thickness. In previous studies, it was shown that coatings with a thickness ≥150 nm showed a more complete surface coverage, resulting in higher antimicrobial activity.^29,40^

**Figure 5:**
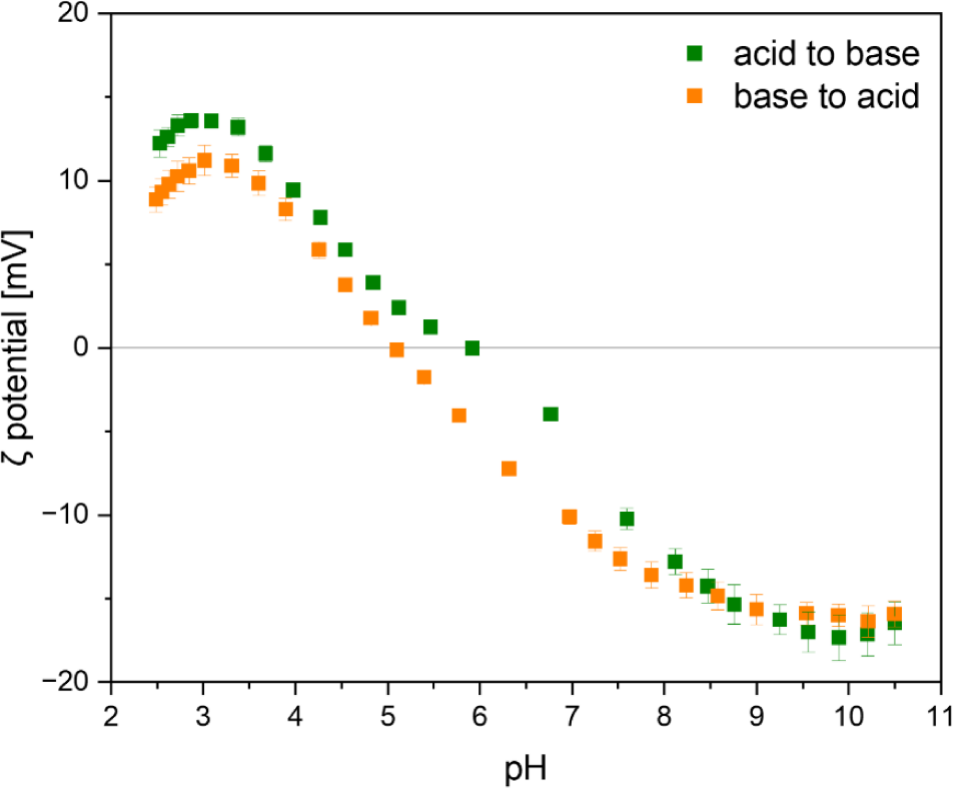
pH-dependent zeta potential of **PNCB2-***co***-BP**. The sample was first titrated from acidic to basic pH (green) and then from basic to acid pH (orange). A shift of the curve of the back titration was observed.

The pH-responsiveness of the **PNCB2-***co***-BP** coatings was demonstrated by electrokinetic measurements, yielding the surface ζ potential as a function of pH value. In that measurement, the curve for **PNCB2-***co***-BP** had the characteristic sigmoidal shape, which is expected for zwitterionic molecules (Figure 5).^20,41^ The results of a titration from acidic to basic pH gave an isoelectric point (IEP) of pH = 5.8 (Table 1). The IEP is the point where the ζ potential of the surface is 0 mV. The ζ potential at pH 7.4 (ζ_phys_), which is relevant for biomedical applications, was −8.25 ± 1 mV, and the p*K*_a_ was 3.8. Thus, the surface ζ potential curve for **PNCB2-***co***-BP** is similar to that of the previously reported **PNCB-***co***-BP** system. Deviations between these systems should not yet be overinterpreted, as these curves constitute two sets of data points only, not a series of polymers from which more solid trends could be deduced. The back-titration curve of the same sample from basic to acidic pH was slightly shifted. To understand this behavior, the chemical stability of the polymer was further investigated.

**Table 1:** Summary of the Electrokinetic Measurement Data Obtained for **PNCB2-co-BP**, in Comparison to Literature Results for **PNCB-co-BP**.^20^^a^

Polymer	IEP [pH]	ζ_max_ [mV]	ζ_phys_ [mV]	ζ_min_ [mV]	p*K*_a_
**PNCB2-***co***-BP**^a^	5.8 ± 0.1	18 ± 5	-8.25 ± 1	-23 ± 6	3.8 ± 0.3
**PNCB-***co***-BP**	5.2 ± 0.1	30 ± 5	-35 ± 5	-41 ± 5	4.1 ± 0.2

a Titration starting at pH 2.5 and ending at pH 10.5.

### Chemical Stability

The stability of the **PNCB2-***co***-BP** networks against hydrolysis was studied over 28 days via transmission IR spectroscopy to monitor the chemical changes and with ellipsometry and AFM to detect changes in the thickness and morphology. For this, polymer coatings were either stored under ambient conditions or immersed into buffer solutions (pH 4.5, 7.4, and 8) at room temperature. The ellipsometry and AFM data are summarized in Table 2.

**Table 2:** Thickness and Roughness Changes of the **PNCB2-co-BP** Network before and after Stability Test for 28 Days in Different Conditions

	Layer thickness [nm]			Roughness^a^[nm]		Contact angle after storage^b^[deg]		
Storage conditions	Before	After	Difference	Before	After	Static^c^	Adv.^d^	Rec.^e^
Air	237.9 ± 0.3	215 ± 1.9	22.9 ± 3.5	0.4 ± 0.0	0.5 ± 0.0	50 ± 1	49 ± 1	44 ± 4
pH 4.5	234 ± 0.5	202 ± 2.1	31.9 ± 3.9	0.4 ± 0.6	1.8 ± 0.4	78 ± 1	78 ± 7	72 ± 7
pH 7.4	243.3 ± 0.5	212.5 ± 1.3	30.8 ± 0.6	0.4 ± 0.0	16.4 ± 7.3	70 ± 2	75 ± 3	68 ± 5
pH 8	224.6 ± 0.4	169.2 ± 0.7	55.4 ± 2.3	0.3 ± 0.0	4.5 ± 0.7	82 ± 3	86 ± 2	76 ± 4

a Error = standard deviation rounded to one decimal point.

b Before stability testing, the contact angle was 54 ± 2° (static), 65 ± 3° (advancing), and 38 ± 5° (receding).

c Laplace–Young model.

d Elliptic model.

e Tangent model.

The data show a thickness loss for all samples, including the one stored in air. As it is highly improbable that this is due to the evaporation of a polymer network component (the sample being carefully dried before each measurement), the effect could be related to the relaxation of residual strains of the structure during storage. Only at pH 8, the thickness loss is substantially higher than for the other conditions, indicating a significant loss of material. After storage in air, the roughness increase is negligible, while a pronounced roughness increase (due to repeatedly drying and reswelling the samples to take measurements) is observed for the three aqueous conditions.

**Figure 6:**
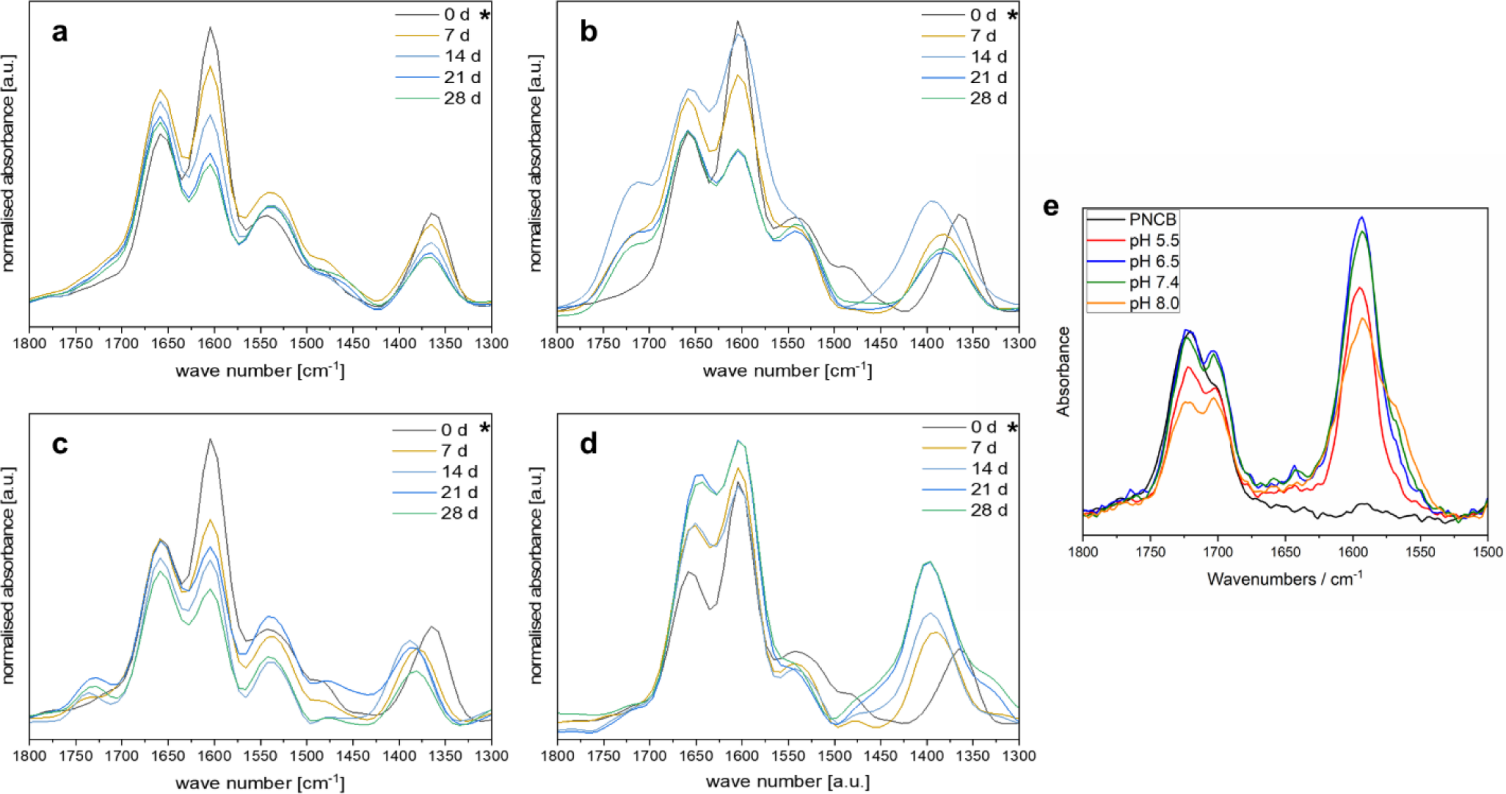
Stability study. IR spectra of the carbonyl region of **PNCB-***co***-BP** after 0–28 days in different storage conditions: (a) air; (b) pH 4.5; (c) pH 7.4; and (d) pH 8. In all figures, the spectrum after storage in air for 0 days (*), representing the untreated sample, is given as a reference. For comparison, the stability study of **PNCB-***co***-BP** is shown (e) (reproduced with permission from ref^20^ copyright The Authors, 2021).

Figure 6 summarizes the recorded IR spectra, focusing on the carbonyl region. The full spectra can be found in the (Figures S25–S28). For a semiquantitative interpretation, the spectra intensities were normalized by adjusting the peak intensity of the ν_(C–H)_ region from 2800 to 3100 cm^–1^ to the same height. Since IR spectra intensities can already deviate when the light is scattered differently within a sample, when the film thickness at the point of measurement varies,^42^ and when the samples are repeatedly removed and reimmersed in the solution, the data will only give a rough trend, not absolute values. As expected, the positions of the stretching vibration bands ν_s(N–H)_ and ν_s(O–H)_ from 3100 to 3660 cm^–1^ change in the studies performed in buffer due to hydration of the networks.^35,43^ The stretching band ν_s(C–H)_ shows small variations in intensity in all cases, but only in the series of spectra taken after storage at pH 8, a systematic decrease of the intensity of the 3027 cm^–1^ band is observed, while that of the band at 2857 cm^–1^ increases in parallel. This gives reason to assume that a significant change in the chemical structure of the polymer occurred.^35^

Due to the comparatively low cross-linker content, the signals originating from the cross-linker units will be neglected in the following discussion. In Figure 6, it can be seen that all networks recorded in transmission mode show the characteristic stretching vibration bands ν_s(C=O)_ of amide I (∼1660 cm^–1^)^44^ and of amide II (∼1530 cm^–1^),^35^ as well as the asymmetric stretching band ν_as(C=O)_ (∼1600 cm^–1^) and the corresponding symmetric stretching band ν_s(C=O)_ (∼1360 cm^–1^) characteristic of the carboxylate. In the ATR IR spectra of the dry polymer powder (Figure 2), the ν_s(C=O)_ band of carboxylic acid (∼1700 cm^–1^)^35^ was found, which is characteristic of the polymer in its cationic state. In the surface-attached polymer films, this band was replaced by the ν_as(C=O)_ band (∼1600 cm^–1^) and the corresponding ν_s(C=O)_ band (∼1360 cm^–1^) of the carboxylate. This can be explained by the dissolution of the polymer for spin-coating in TFE that contained sufficient water to allow proton exchange. In Figure 6a summarizing the IR spectra of the samples stored in air, an initial increase of the amide I and II bands is observed after 7 days, followed by a decrease of these bands back to the level of the dry sample over 28 days. The carboxylate bands slightly decreased within 28 days. Notably, there is no shift in ν_s(C=O)_ band of the carboxylate since all samples were measured in the dry state. Since no substance loss in that time span is expected during dry storage, and hydrolysis of the amide band would lead to a carboxylate increase instead of the observed decrease, these changes are assumed to be related to the systematic variations of IR measurements discussed above, as well as to the thickness decrease of the sample due to molecular relaxation, which was observed by ellipsometry.

In Figure 6b summarizing the spectra of samples stored in buffer at pH 4.5, a similar pattern is observed, indicating no substantial hydrolysis. The most important change between Figure 6a and this series is the development of a shoulder around 1715 cm^–1^, which is due to the formation of a carboxylic acid group by partial protonation of the carboxylate at pH 4.5. Also, in this figure and all that follow, the ν_s(C=O)_ band of the carboxylate shifts to slightly higher wavenumbers (about 1390 cm^–1^), possibly due to hydrogen bonding with residual water in these samples. Again, the carboxylate peak decreases while there is only a slight difference in intensity between the amide signals of the dry sample and the sample after 28 days of incubation in buffer.

Figure 6c shows the series of measurements obtained at pH 7.4, which is the most relevant for applications. Here, the additional carboxylic acid (1731 cm^–1^) peak also emerged, but to a lesser extent, which is in line with the expectation that the carboxylate gets less protonated in physiological conditions than at acidic pH. Otherwise, a slightly stronger decrease of the amide bands is observed than in (a) and (b), together with a decrease of the carboxylate bands. Overall, the IR spectra shown in Figure 6a–c are very similar in the intensity pattern of the amide and the carboxylate. In Figure 6d summarizing the spectra of samples stored at pH 8, the relative intensity of the carboxylate peak around 1398 cm^–1^ compared to the other peaks strongly increases. This is expected with a near-complete degree of deprotonation of COOH at that pH, which is also confirmed by the full loss of the signal of the carboxylic acid peak.

At the same time, the intensity of the amide I signal around 1655 cm^–1^ increases to a similar intensity as the peak of the carboxylate group. The substantial increase of the carboxylate relative to the amide could indicate the formation of more carboxylate due to amide hydrolysis. This is also consistent with the highest thickness loss of the samples stored at pH 8 over time. This is unexpected due to the known hydrolysis stability of amides, which typically require harsher conditions such as higher temperatures and stronger acids or strong bases for hydrolysis.^45−47^ Also, it is not quite clear why the normalized intensity of the amide increases over time rather than decreases. Further investigations will focus on understanding this behavior to rule out other side reactions such as Hofmann elimination on the quaternary nitrogen group, degradation of the polymer backbone, or simply the failing of the silane prefunctionalization of the silicon substrate.

Overall, in comparison to previously studied **PZI** and **PNCB-***co***-BP**, a significant stability improvement can be noted. While these polymers start to hydrolyze after just 1 day in aqueous media,^20^ and a strong degree of hydrolysis of the ester groups was detected under physiological conditions after 22 days (Figure 6e),^20^ in **PNCB2-***co***-BP** the amide group does not disappear even under mildly acidic or basic conditions and is only slightly affected by storage at physiological pH for 28 days. This constitutes a significant improvement in chemical stability and is a further important step toward applications of these systems.

AFM images of the samples after 28 days of the respective storage conditions (Figures S21–24) indicate that the observed increase in roughness is due to particles accumulated on the surfaces—most likely residual salts from the different buffers. Contact angle measurements showed that the hydrophilicity of the surfaces was not much affected by storage in air but was significantly reduced after storage in buffer (Table 2). This is consistent with both partial hydrolysis of the amide bonds and general molecular rearrangements of the samples to minimize the surface energy.

### Antimicrobial Activity

Antimicrobial activity studies were performed on **PNCB2-***co***-BP** networks using a simplified version of the Japanese Industrial Standard JIS Z 2801 (corresponding to ISO 22196) for testing the antimicrobial activity of technical products and nonporous surfaces.^10^*Escherichia coli* (DSM498) was used as a test organism. The previously reported **PZI** was used as a control due to its excellent antimicrobial activity.^15^

**Figure 7:**
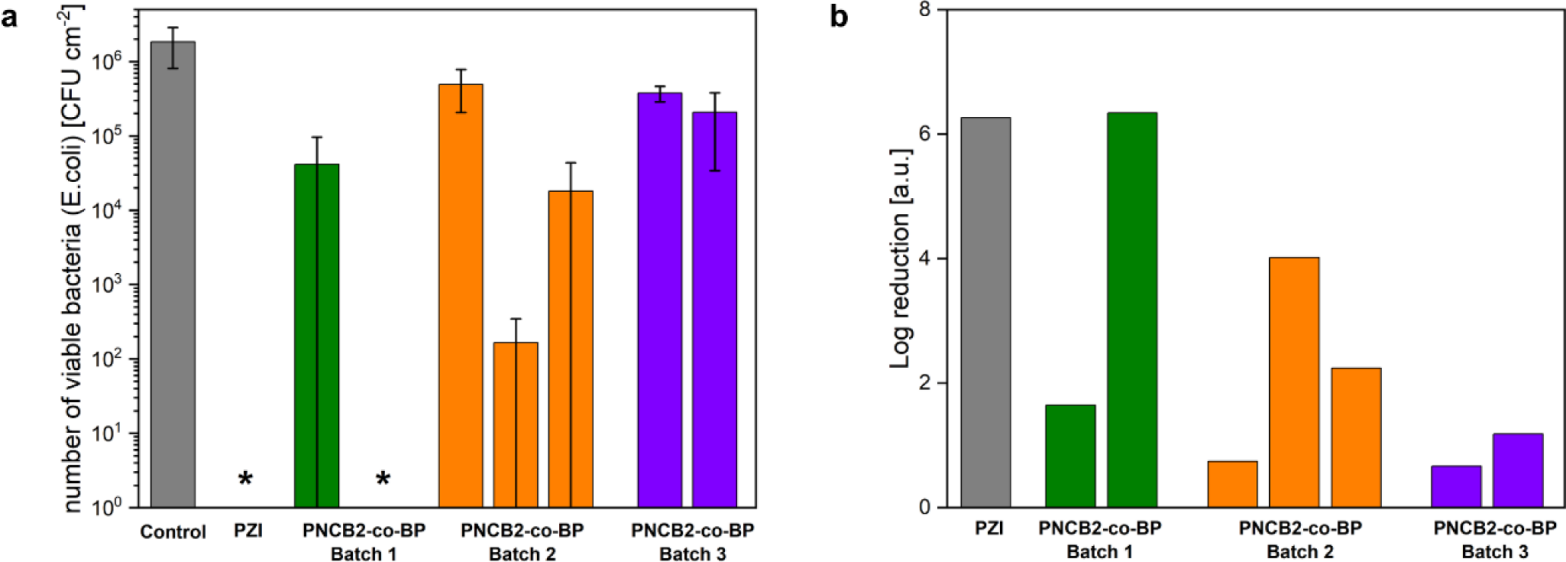
Microbiological characterization of different batches of **PNCB2-***co***-BP**. Silicium wafers were used as an untreated control. **PZI** was used as control to demonstrate antibacterial action. (a) The number of viable bacteria recovered from the test and control specimen after 24 h was quantified. * points out the samples, where CFUs were not detected. (b) Based on the data in (a), the antibacterial activity was expressed as log reduction against the growth control (b).

In Figure 7, the results of seven independent antimicrobial experiments using **PNCB2-***co***-BP** obtained from three different synthesis batches are summarized. The results are inconclusive. This is neither a result of irreplicable coating quality nor poor reproducibility of the antibacterial assay. A thorough analytical control of the coatings indicated that they were of good quality, and the controls and samples tested side-by-side in the antimicrobial assay indicated that the assay worked well with all other materials. Two runs confirmed a high to very high antimicrobial activity (4–6 log reduction), whereas in other cases, the log reduction was only 1–2. It is possible that this result is due to the high growth rate of the bacteria within 24 h (from 10^4^ bacteria to 10^6^ bacteria on the growth control). While the **PZI** control is consistently able to clear that bacterial load, it is too much for **PNCB2-***co***-BP**, depending on the exact experimental conditions in that run. Overall, the data imply that **PNCB2-***co***-BP** exhibits antimicrobial activity, but to a lesser extent than **PZI**. Further studies, also with different bacteria, are needed to exactly quantify the antimicrobial activity of the material. This will hopefully explain the unusual lack of reproducibility in this assay, which may be related to the exact switching point of the charge-switching properties of the material.

## Discussion

While the chemical stability of polymers is generally analogous to that of their organic small molecule counterparts, there are important examples when that analogy fails. Ester groups in polymers constitute such a case. The stability of an ester group in a polymer toward hydrolysis depends not only on the intrinsic reactivity of that particular ester but also on the overall hydrophobicity and the degree of crystallinity of the polymer.^48^ This is well-known in the field of degradable polymers. Thus, hydrophobic polyesters are stable materials that can be spun into fibers for textiles or be used as robust organic glass. In the field of polyzwitterions, the ester group in poly(methacryloyloxylethyl phosphorylcholine) (**polyMPC**) has proven remarkably stable toward hydrolysis despite the overall hydrophilicity of that polymer, which makes the ester group in principle accessible to nucleophilic attack. In fact, **polyMPC** is to date one of the few polyzwitterions that has found its way into applications due to this surprising stability.^49^ It is assumed that the reason for the unexpected stability of **polyMPC** is that its high density of phosphate and ammonium ions leads to a strong cooperative buffering effect. Thus, nucleophiles are neutralized when approaching the carbonyl group.^50^ Assuming a similar behavior for polyzwitterions in general, our previous work also focused on polyzwitterions containing ester groups,^15,19,20^ as the synthesis of such polymers was simpler than that of the corresponding structures with more stable linkers (e.g., ethers or amides). When early systems like **PZI** were not hydrolytically stable, we assumed that this was due to the presence of a high density of primary ammonium groups, which could dissociate into amines, leading to transesterification. We replaced those groups with quaternary ones and obtained structures like **PNCB-***co***-BP**. While this was an improvement in stability, IR data showed that these materials also underwent ester hydrolysis after 22 days.^20^ Thus, the hydrolytic stability of **polyMPC** is not simply due to its polyzwitterionic character. When comparing **polyMPC** and polyzwitterionic sulfobetaines to our systems, an important difference is their pH-responsiveness, as demonstrated in the pH-dependency of the zeta potential and the position of the isoelectric point. It can be assumed that these parameters can be correlated to the stability of the ester group toward nucleophiles in such systems, as they indicate how easy it is to create a (nano)environment near the polymer chains that allows nucleophilic attack. Apparently, ester groups in poly(carboxyzwitterions) are much more labile than the corresponding poly(sulfobetaine) and poly(phosphatidylcholine) systems. Thus, to enhance the stability of poly(carboxyzwitterions), which are attractive due to their charge-switching properties, the ester group needs to be replaced. As expected, in the presented system, the amide group enhanced the chemical stability of the structure, particularly in aqueous media. Although not perfect, this system can be considered as an important step in bringing this family of polymers into biomedical applications.

## Conclusion

In this work, polyzwitterionic **PNCB2-***co***-BP** was synthesized from monomers **NCB2** and **BP**. In these building blocks, the functional groups were linked to the polymerizable groups via amide bonds, so that the target polymer would have increased chemical stability compared to known charge-switchable polymer systems. The monomers readily underwent ROMP with Grubbs third-generation catalyst under ambient conditions, yielding **PNCB2-***co***-BP** with a molar mass around 70,000 g mol^–^.^1^ Surface-attached polymer networks were obtained from this polymer by UV cross-linking. These networks were fully characterized on the molecular and surface properties level. Zeta-potential analysis showed that the network charge can be switched depending on the pH of the surrounding media. Stability studies showed that the amide groups in **PNCB2-***co***-BP** substantially improved the stability against hydrolysis compared to the corresponding ester-based systems. Only under basic conditions did the network show partial hydrolysis. The antimicrobial properties need to be studied in more detail in future work. Likewise, cell compatibility and protein adsorption studies will be performed to complement these data so that a full bioactivity profile of this material becomes available and its usefulness for future applications can be assessed.

## Supporting Information Available

Details about the materials used (Section SI1), NMR and mass spectra of the molecules synthesized (Section SI2), AFM images of the surfaces after the stability studies (Section SI3), and the full transmission IR spectra as well as zoom-ins of the C–H regions (Section SI4) (PDF)
