# Supplementary material for: Reversibly Charge-Switching Polyzwitterionic/Polycationic Coatings for Biomedical Applications: Optimizing the Molecular Structure for Improved Stability
Source: Langmuir. 2025 Mar 5;41(10):6644–56. doi: 10.1021/acs.langmuir.4c04358 (PMC11924213; doi:10.1021/acs.langmuir.4c04358)
Supplement: Supplementary file 1 — la4c04358_si_001.pdf [file la4c04358_si_001.pdf]

Supporting Information

Reversibly Charge-Switching Polyzwitterionic/Polycationic Coatings for Biomedical Applications: Optimizing the Molecular Structure for Improved Stability.

*Sophie H.E. Schneider,[1,2] Kathrin Lehnert,[1,2] Marie A. Thome,[1,2] Annette Kraegeloh,[3] Karen Lienkamp[1,2, *]*

[1] Chair for Polymer Materials, Department of Materials Science & Engineering, Saarland University, Campus C4 2, 66123 Saarbrücken, Germany.

[2] Saarland Center for Energy Materials and Sustainability (Saarene), Saarland University, Campus, Campus C4 2, 66123 Saarbrücken, Germany.

[3] INM-Leibniz Institute for New Materials, Campus D2 2, 66123 Saarbrücken, Germany.

* E-mail: karen.lienkamp@uni-saarland.de

Table of Contents

SI1 – Materials

SI2 – Analytical Data of Monomers and Polymers

SI3 – AFM Images

SI4 – Stability Study – Transmission IR Spectra

SI1 - Materials

All chemicals were received as stated in the following: ethyl actetate (EA, > 99.5 %), n-hexane (>99.0 %) diethyl ether (≥ 99.5 %), aqueous solution of sodium hydroxid (NaOH 0,05 mol L^{-1} and 0.1 mol L^{-1}) and aqueous solution of hydrochloric acid (HCl, 0,05 mol L^{-1} and 0.1 mol L^{-1}) from Carl Roth (Karlsruhe, Germany); 2,2,2-trifluororethanol (TFE, 99.9 %), β-Propiolactone (97 %) from ABCR (Karlsruhe, Germany), 4-dimethylaminopyridine (DMAP, >99.0 %), *N,N*-dimethyl-ethylenediamine (>98.0 %) from TCI (Eschborn, Germany); ethyl vinyl ether (EVE, 99 %), HCl (4.0 mol L^{-1} in dioxane), exo-bicyclo(2.2.1)hept-5-ene-2-carboxylic acid (97 %) from Sigma-Aldrich (Taufkirchen, Germany); 4-aminobenzophenone (95.0 %) from BLD Pharmatech GmbH (Reinbek, Germany); dicyclohexylcarbodimide (DCC, 99.0 %) tetrahydrofuran (THF, ≥ 99.8 % and 98.5 % extra dry) from Thermo Fisher Scientific (Schwerte, Germany); 3-aminopropyltriethxysilan (APTES, 99 %) and dichloromethane (DCM, 99 %) from Fisher Sientific (Nidderau, Germany); Ethanol (EtOH, 99 % with 1% methyl-ethyl-ketone (MEK)) from BCD Chemie GmbH (Friedrichsthal, Germany); toluene (99.8 %) from Stockmeier Chemie (St. Ingbert, Germany) and deuterated solvents $CDCl_3$ (99.95 % with 0.03 % tetramethylsilane TMS) and D_2O (99.95 %) from deutero (Kastellaun, Germany). For anhydrous conditions DCM was stirred over CaH_2 under inert atmosphere and freshly distilled before every usage. Three circles of freeze-pump-thaw were performed for TFE: All over reagents were used as received.

S2 - Analytical Data of Monomers and Polymers

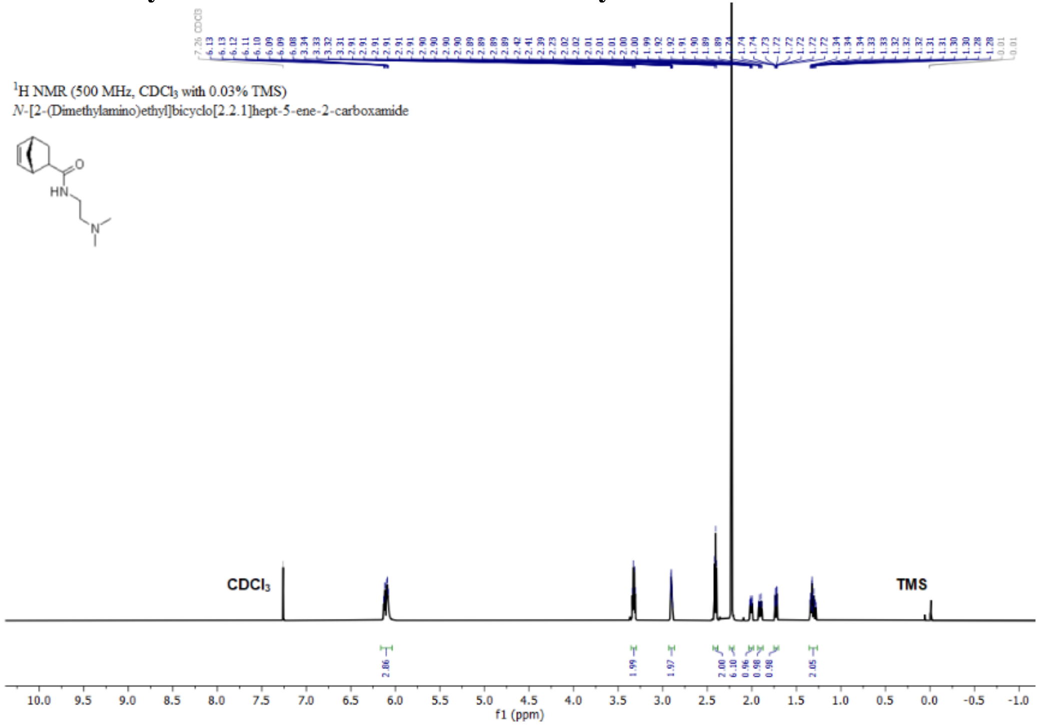

Figure S1 ^{1}H NMR spectrum of N-[2-(Dimethylamino)ethyl]bicyclo[2.2.1]hept-5-ene-2-carboxamide (**2**).

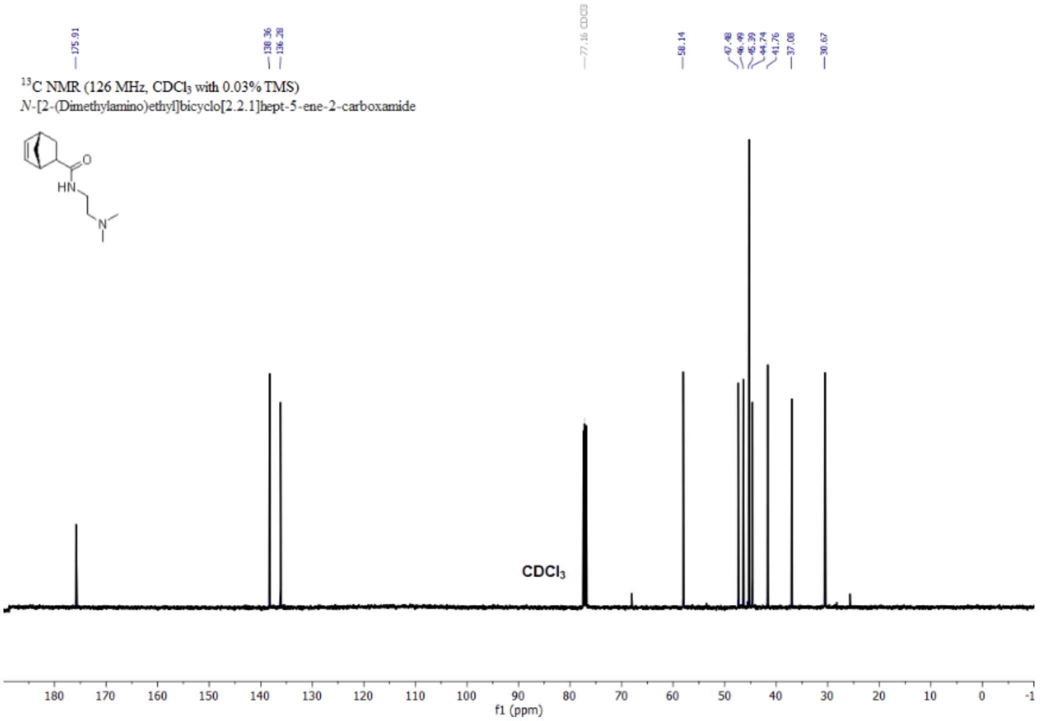

Figure S2 ^{13}C NMR spectrum of N-[2-(Dimethylamino)ethyl]bicyclo[2.2.1]hept-5-ene-2-carboxamide (**2**).

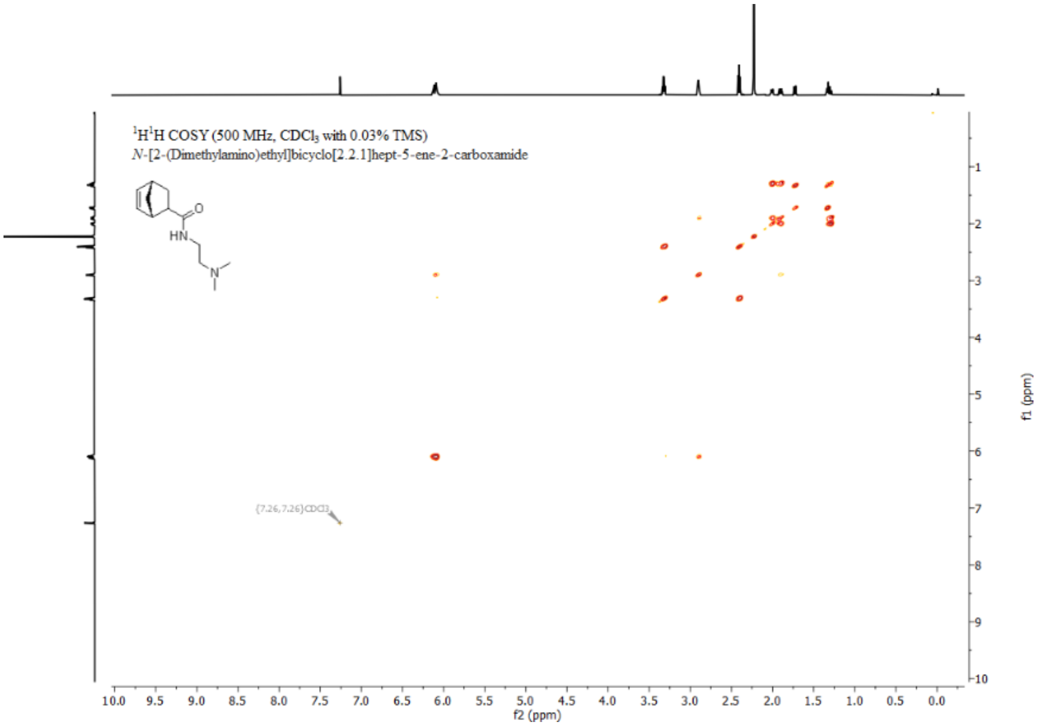

Figure S3 ^{1}H^{1}H COSY NMR spectrum of N-[2-(Dimethylamino)ethyl]bicyclo[2.2.1]hept-5-ene-2-carboxamide (**2**).

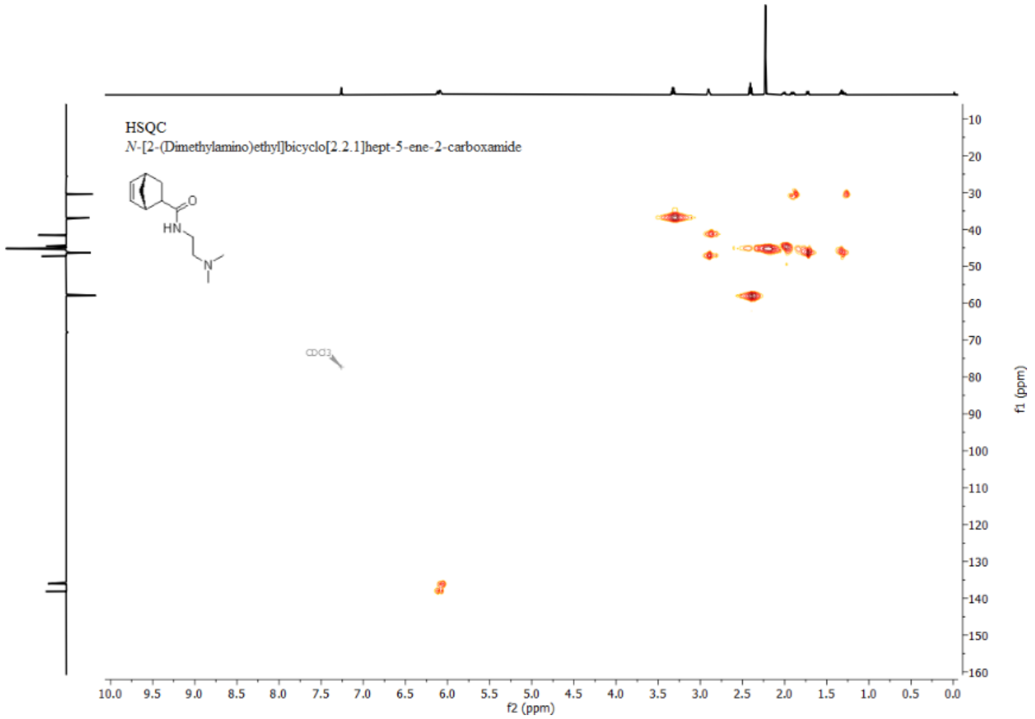

.

Figure S4 HSQC NMR spectrum of N-[2-(Dimethylamino)ethyl]bicyclo[2.2.1]hept-5-ene-2-carboxamide (**2**).

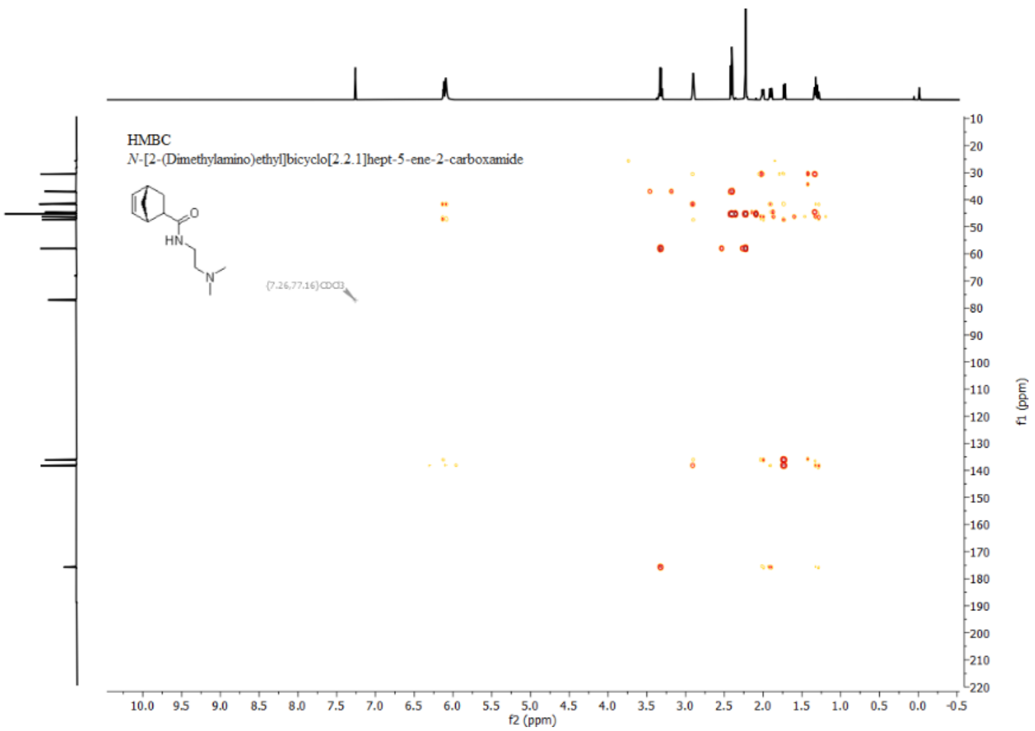

Figure S5 HMBC NMR spectrum of N-[2-(Dimethylamino)ethyl]bicyclo[2.2.1]hept-5-ene-2-carboxamide (**2**).

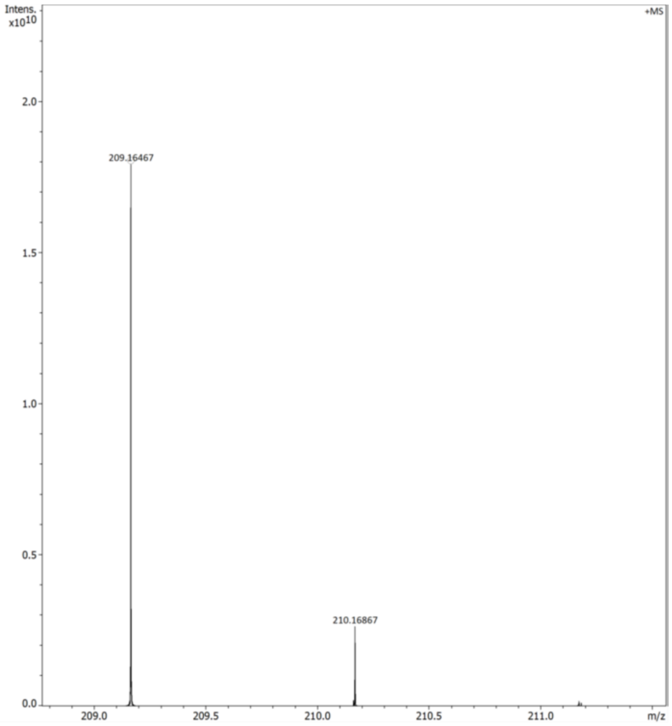

Figure S 6 HRMS ESI mass spectrum of N-[2-(Dimethylamino)ethyl]bicyclo[2.2.1]hept-5-ene-2-carboxamide (**2**).

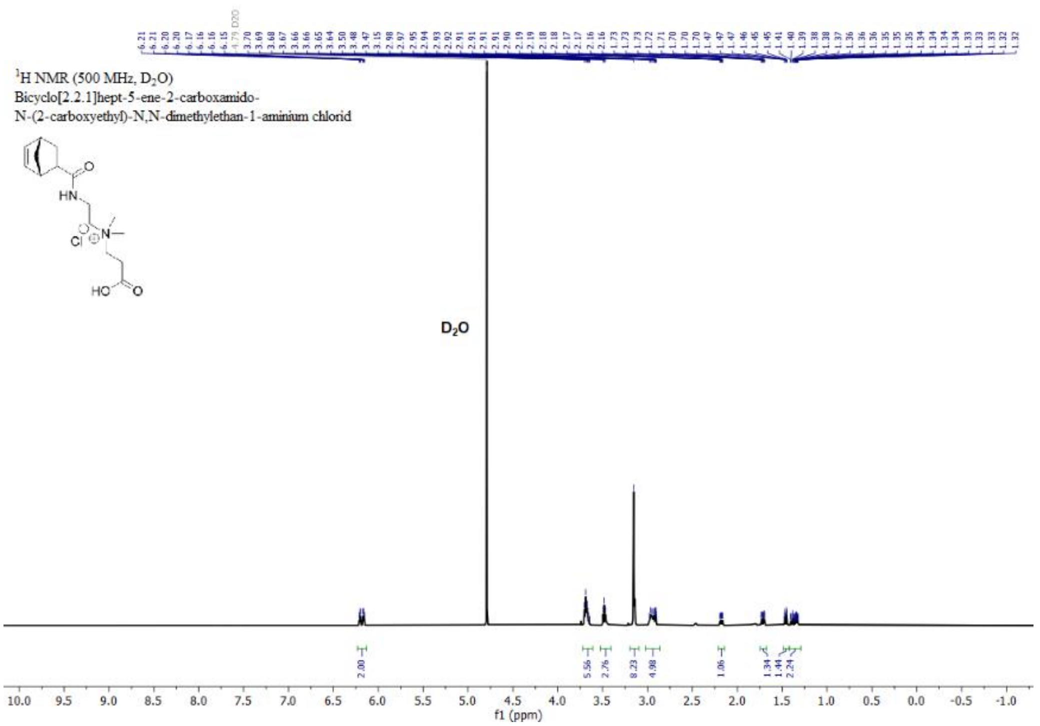

Figure S7 ^{1}H NMR spectrum of Bicyclo[2.2.1]hept-5-ene-2-carboxamido-N-(2-carboxyethyl)-N,N-dimethylethan-1-aminium chloride (**NCB2**).

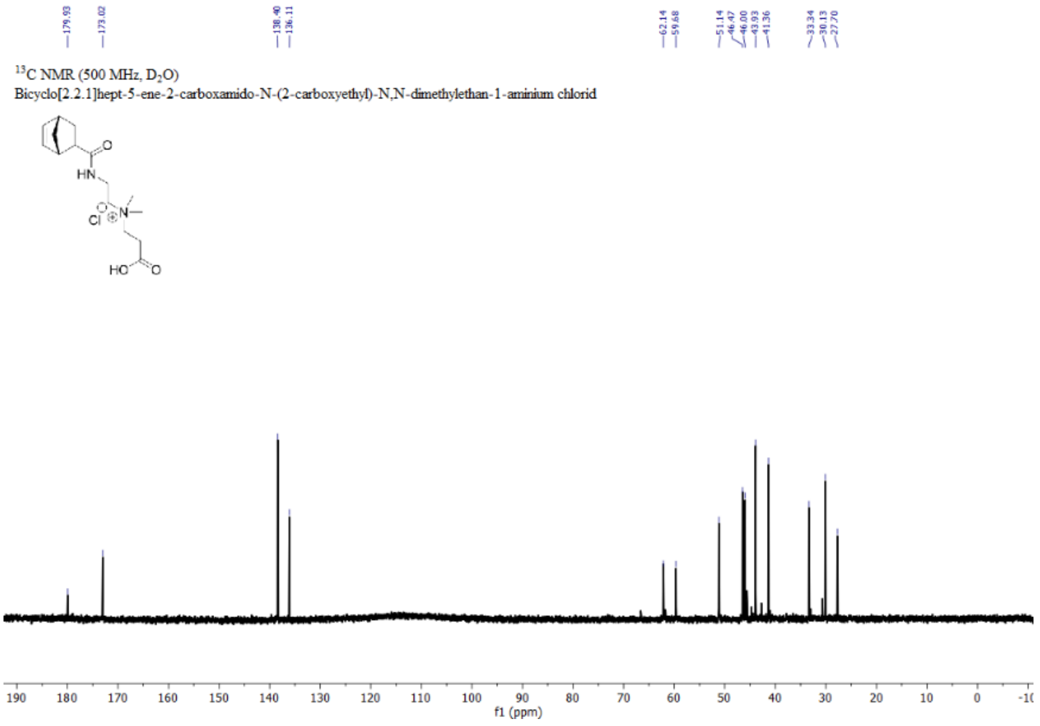

Figure S8 ^{13}C spectrum of Bicyclo[2.2.1]hept-5-ene-2-carboxamido-N-(2-carboxyethyl)-N,N-dimethylethan-1-aminium chloride (**NCB2**).

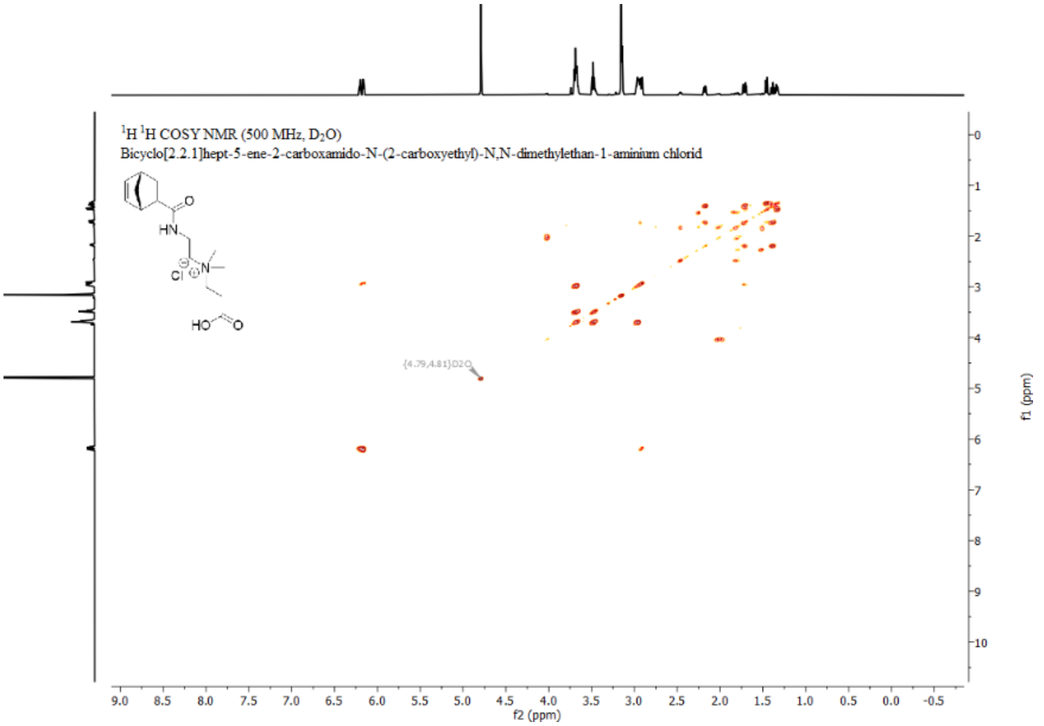

Figure S9 ^{1}H^{1}H COSY spectrum of Bicyclo[2.2.1]hept-5-ene-2-carboxamido-N-(2-carboxyethyl)-N,N-dimethylethan-1-aminium chloride (**NCB2**).

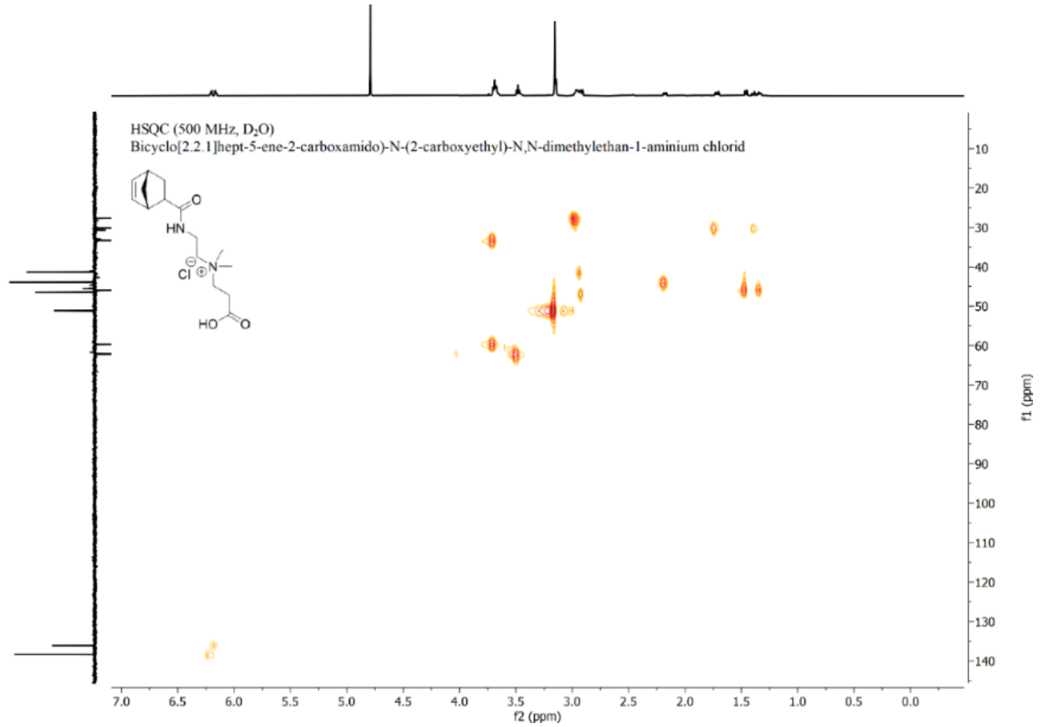

Figure S10 HSQC NMR spectrum of Bicyclo[2.2.1]hept-5-ene-2-carboxamido-N-(2-carboxyethyl)-N,N-dimethylethan-1-aminium chloride (**NCB2**).

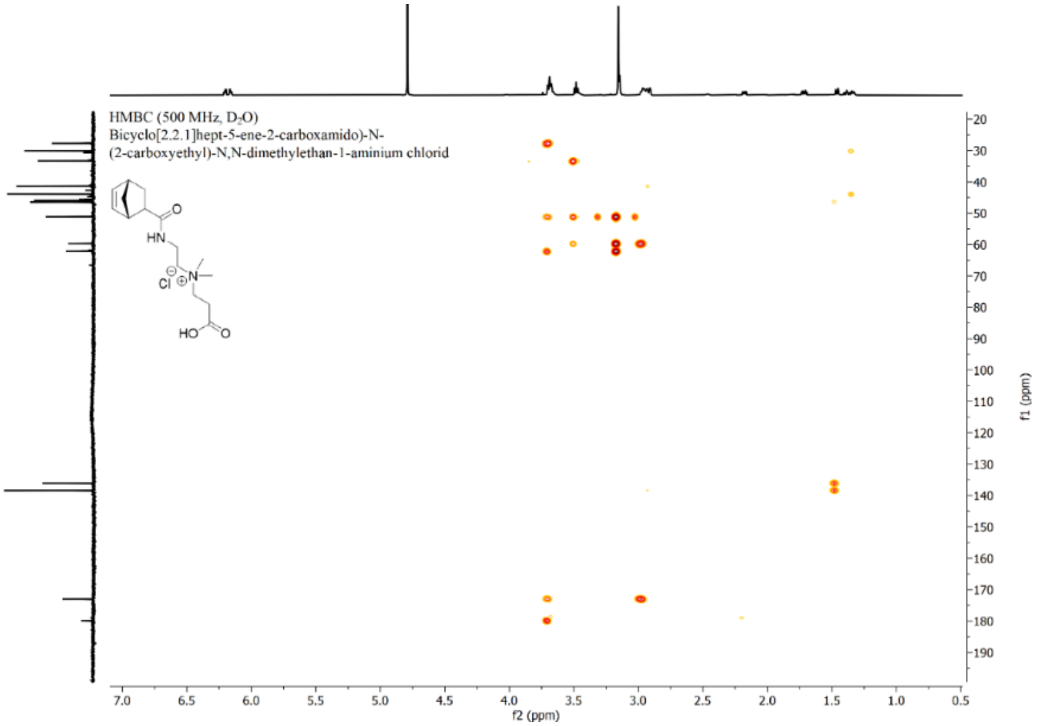

Figure S11 HMBC NMR spectrum of Bicyclo[2.2.1]hept-5-ene-2-carboxamido-N-(2-carboxyethyl)-N,N-dimethylethan-1-aminium chloride (**NCB2**).

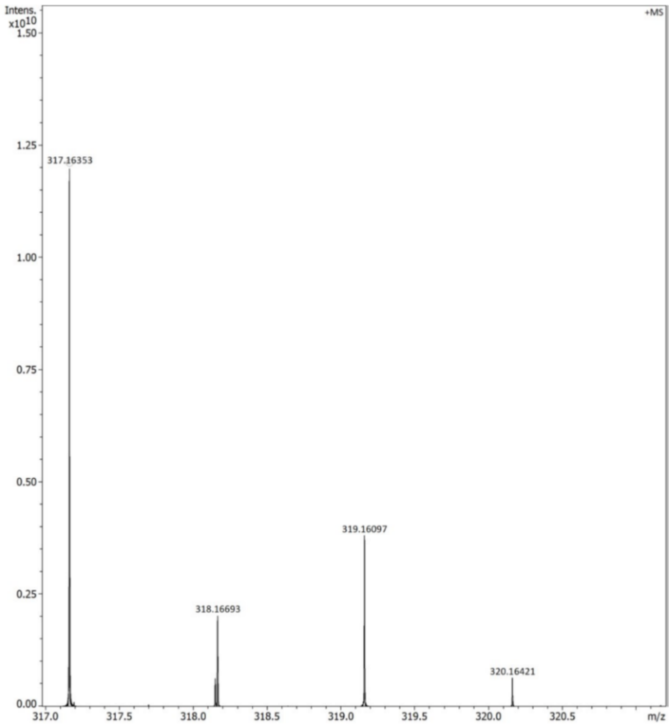

Figure S 12 HRMS ESI mass spectra of Bicyclo[2.2.1]hept-5-ene-2-carboxamido-N-(2-carboxyethyl)-N,N-dimethylethan-1-aminium chloride (**NCB2**).

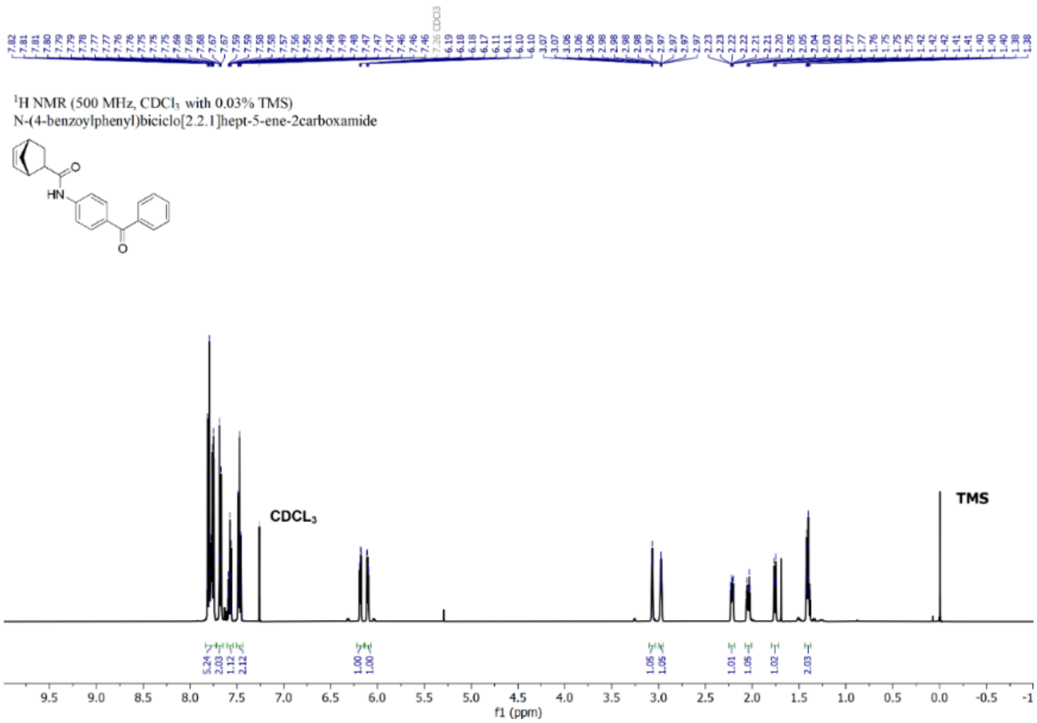

Figure S 13 ^{1}H NMR spectra of N-(4-benzoylphenyl)bicyclo[2.2.1]hept-5-ene-2-carboxamide (**BP**).

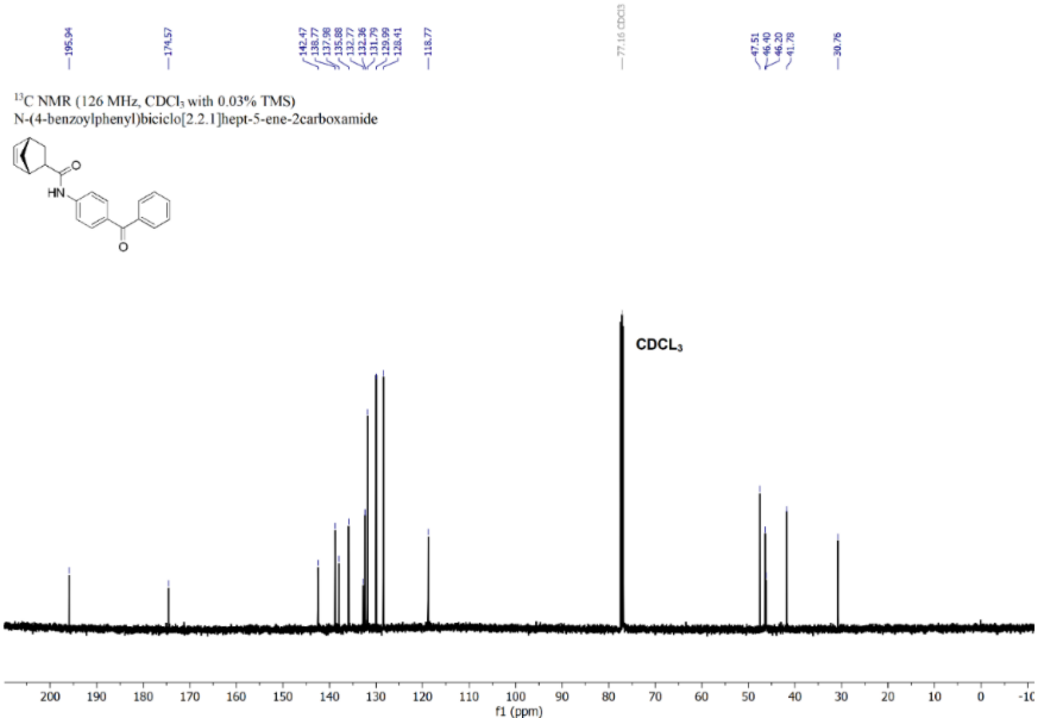

Figure S 14 ^{13}C NMR spectra of N-(4-benzoylphenyl)bicyclo[2.2.1]hept-5-ene-2-carboxamide (**BP**).

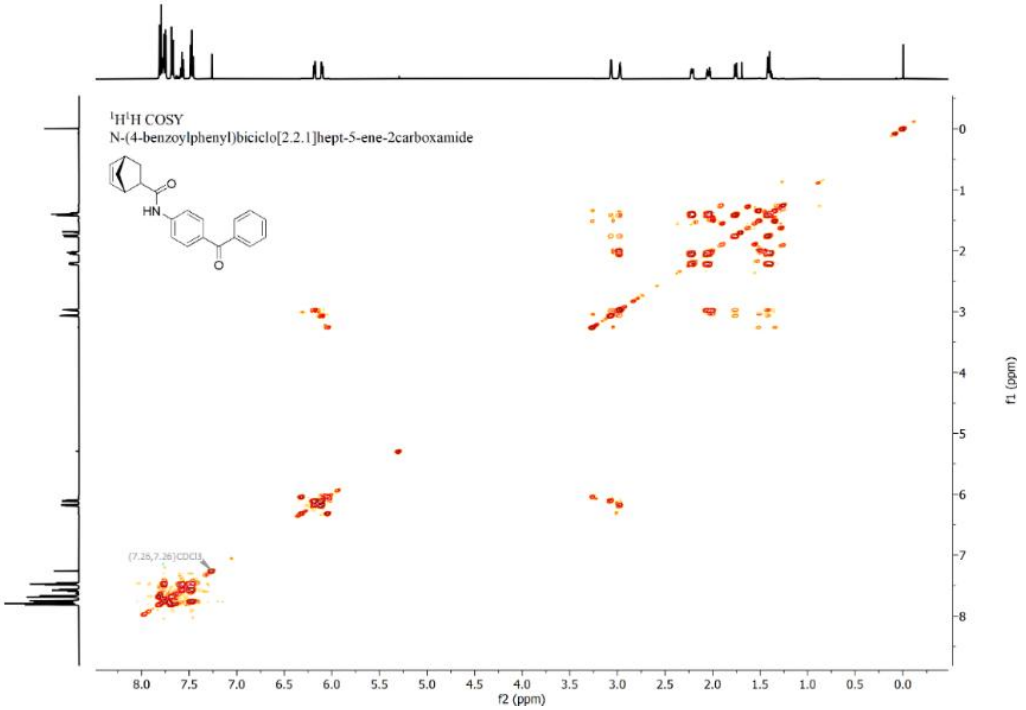

Figure S 15 $^{1}H^{1}H$ COSY NMR spectra of N-(4-benzoylphenyl)bicyclo[2.2.1]hept-5-ene-2-carboxamid (**BP**).

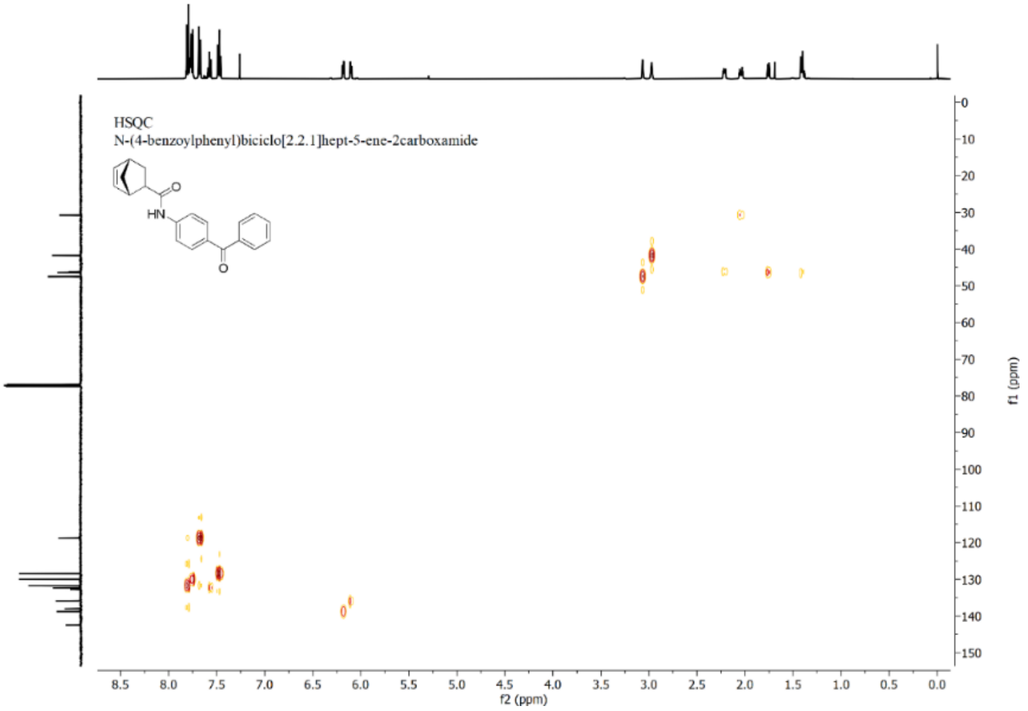

Figure S 16 HSQC NMR spectra of N-(4-benzoylphenyl)bicyclo[2.2.1]hept-5-ene-2-carboxamide (**BP**).

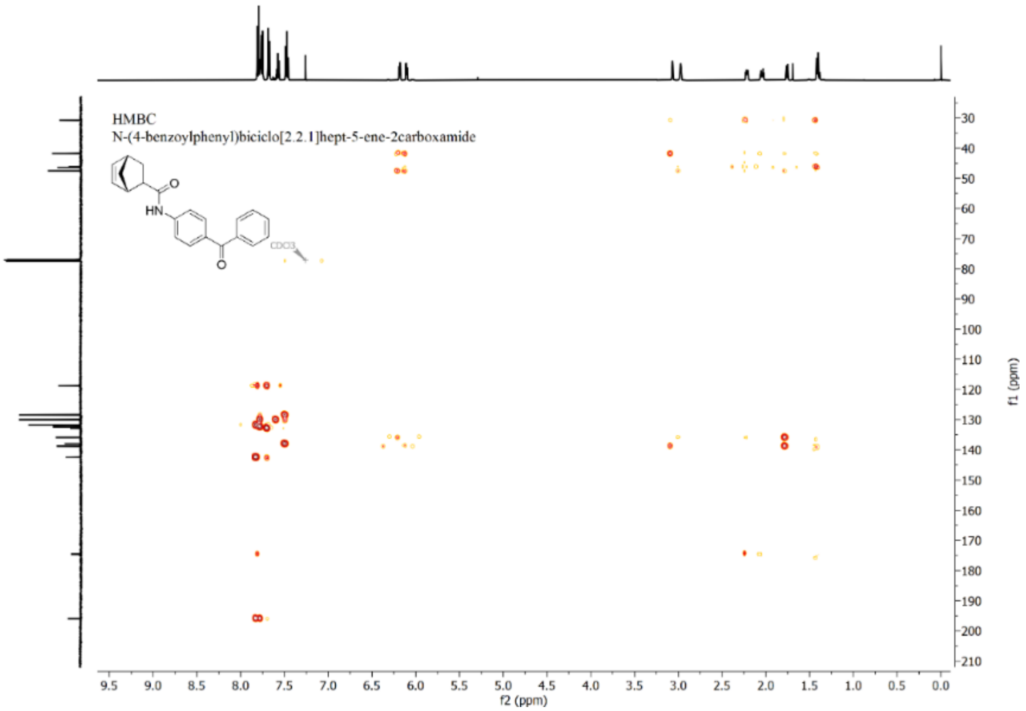

Figure S 17 HMBC NMR spectra of N-(4-benzoylphenyl)bicyclo[2.2.1]hept-5-ene-2-carboxamide (**BP**).

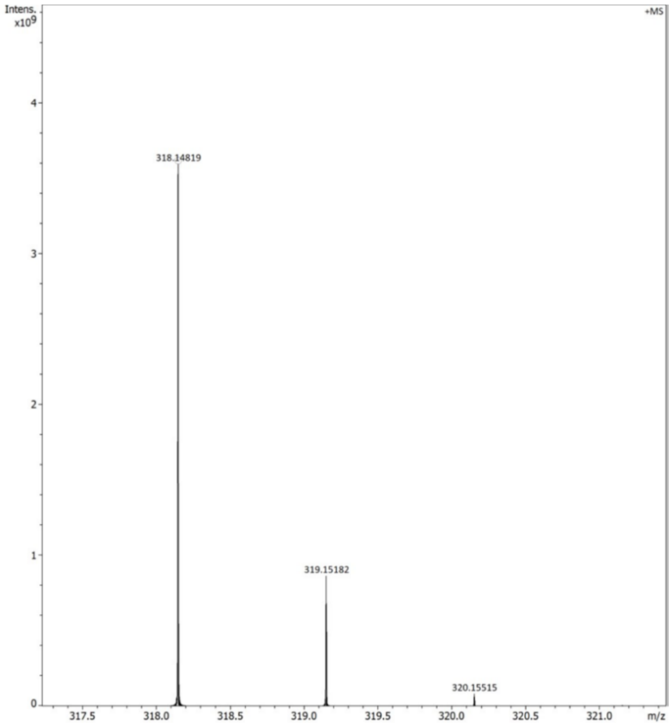

Figure S 18 HRMS ESI mass spectra of N-(4-benzoylphenyl)bicyclo[2.2.1]hept-5-ene-2-carboxamide (**BP**).

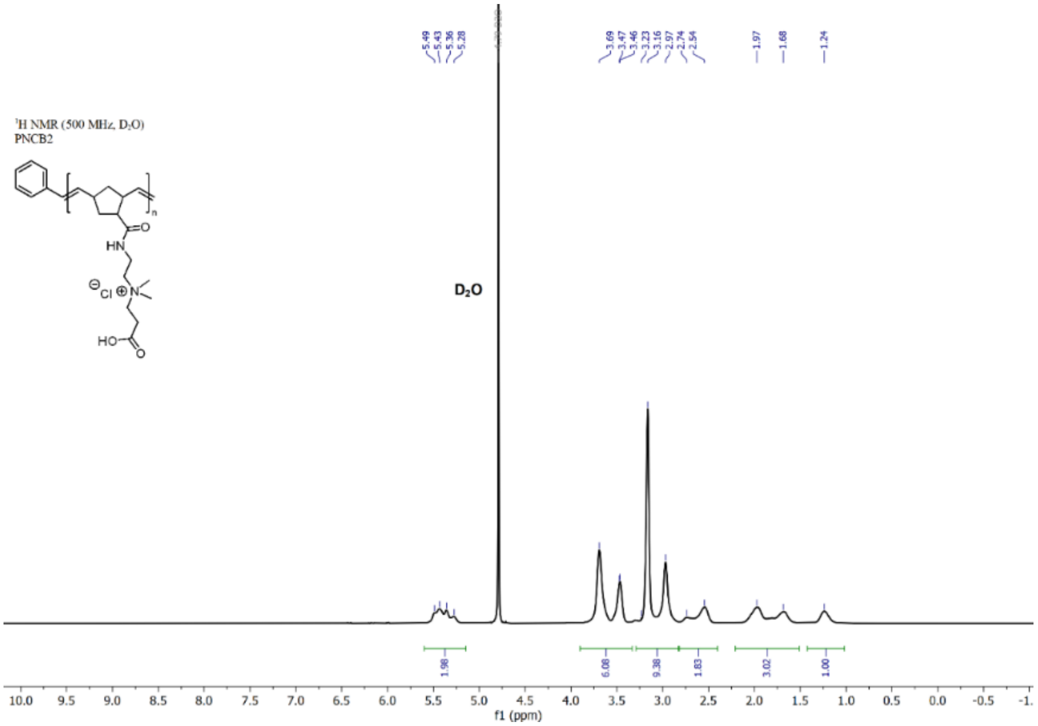

Figure S19 ^{1}H NMR spectrum of **PNCB2**.

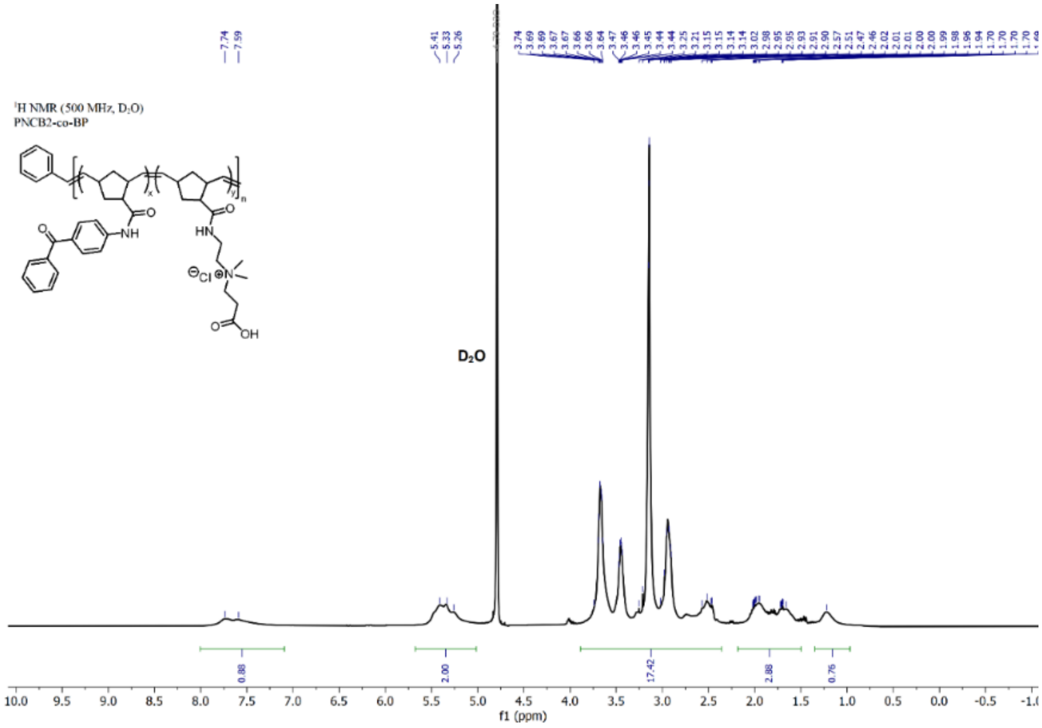

Figure S20 ^{1}H NMR spectrum of **PNCB2-co-BP**.

SI 3 – AFM images

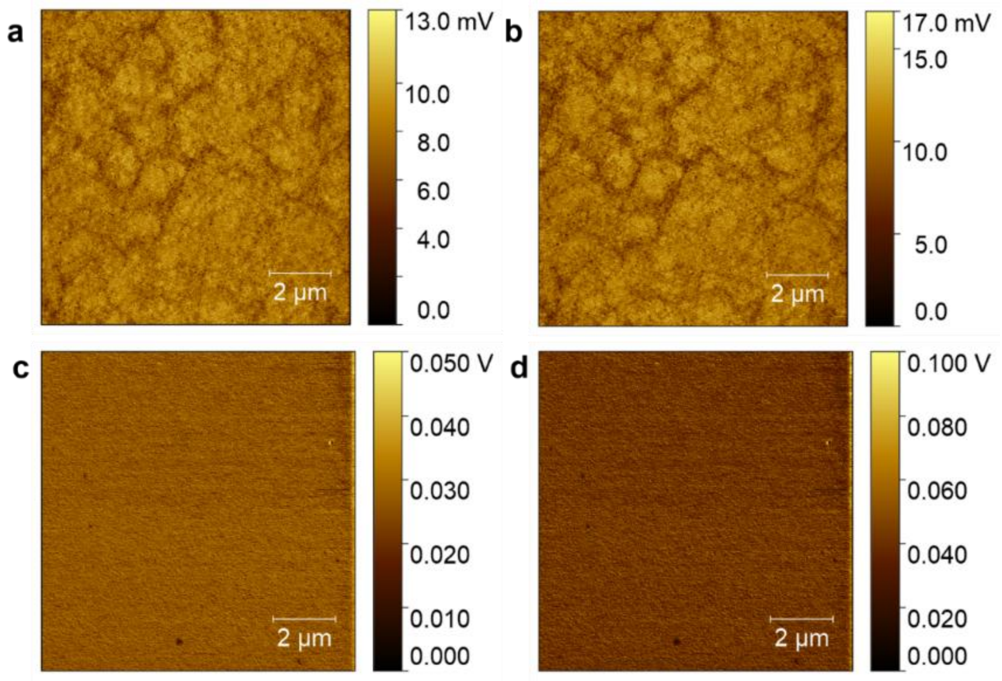

Figure S21 AFM images of **PNCB2** (a: Inphase, b: Quadrature) and **PNCB2-co-BP** (c: Inphase, d: Quadrature)

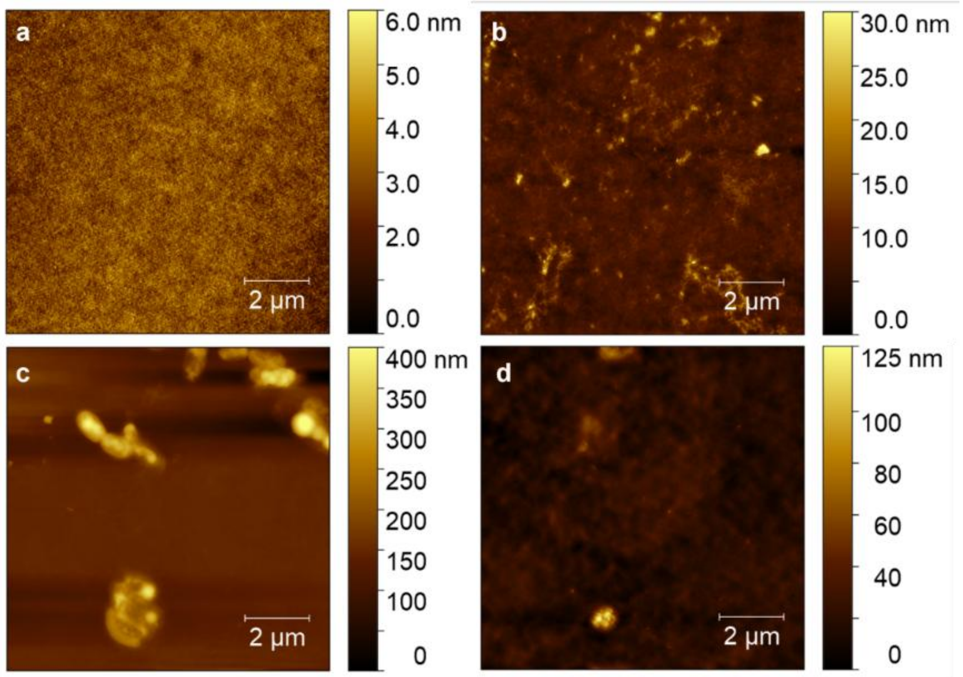

Figure S22 AFM height sensor image of **PNCB2-co-BP** networks after stability study (air (a), pH 4 (b), pH 7.4 (c) and pH8 (d)).

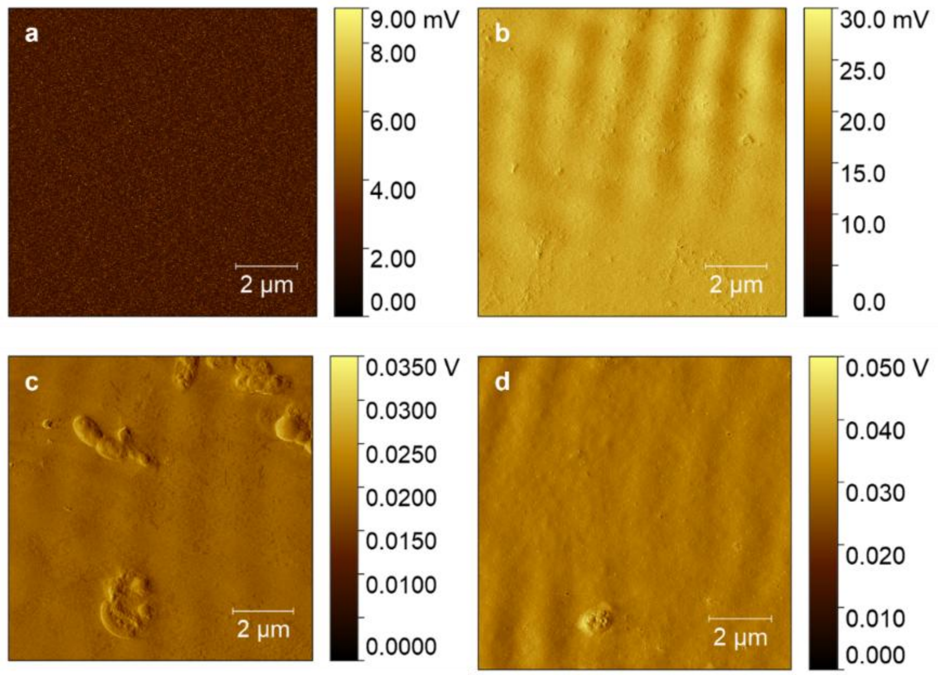

Figure S23 AFM inphase image of **PNCB2-co-BP** networks after stability studie (air (a), pH 4 (b), pH 7.4 (c) and pH8 (d)).

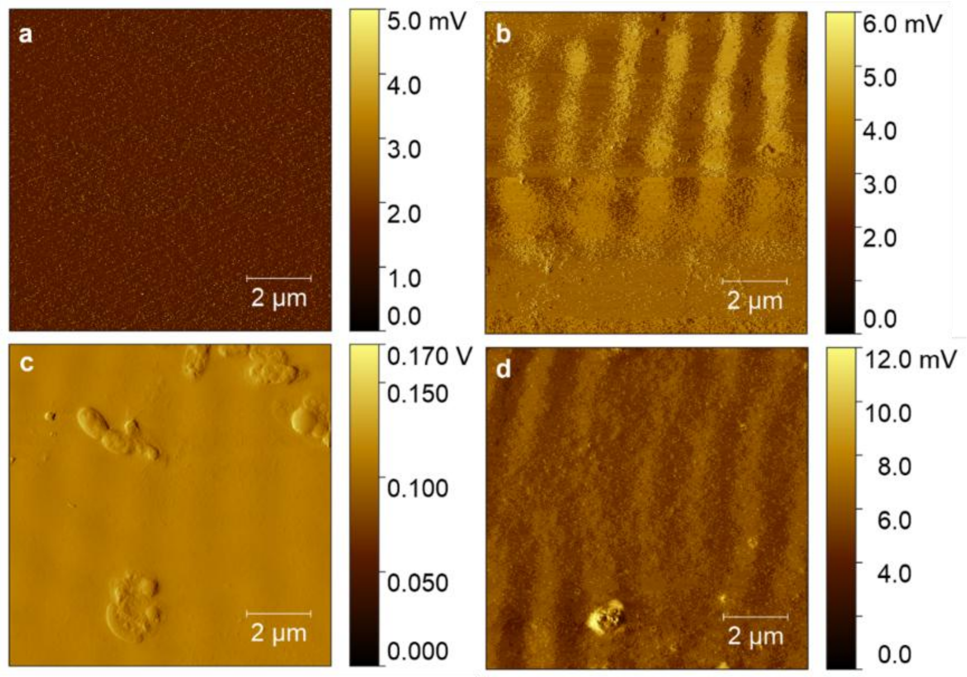

Figure S24 AFM quadrature image of **PNCB2-co-BP** networks after stability studie (air (a), pH 4 (b), pH 7.4 (c) and pH8 (d)).

S4 – Stability study – Transmission IR spectra of coated silicon wafers.

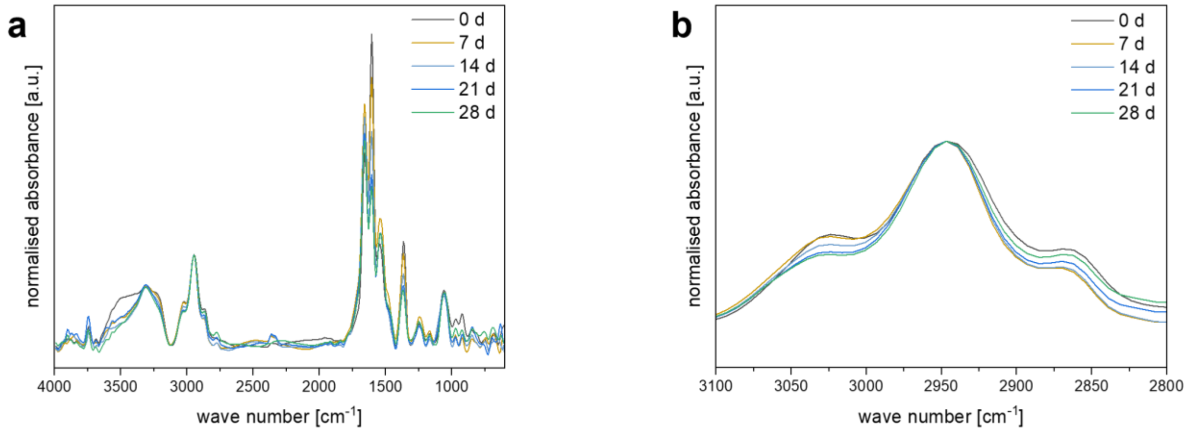

Figure S25 Stability study via FTIR spectra of **PNCB2-co-BP** networks stored on air over 28 days. The full spectra (a) and the zoom into the C-H stretching region (b).

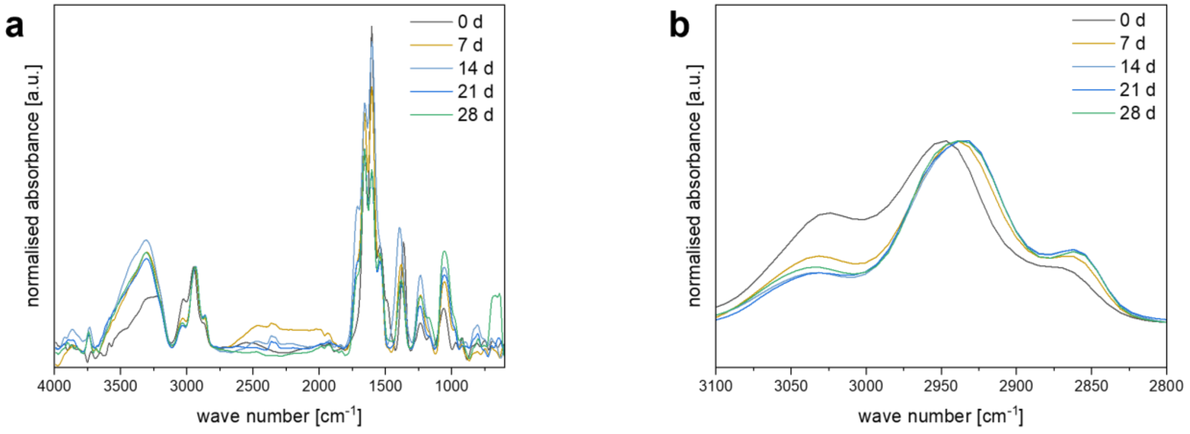

Figure S26 Stability study via FTIR spectra of PNCB2-co-BP networks stored at pH 4.5 over 28 days. The full spectra (a) and the zoom into the C-H stretching region (b).

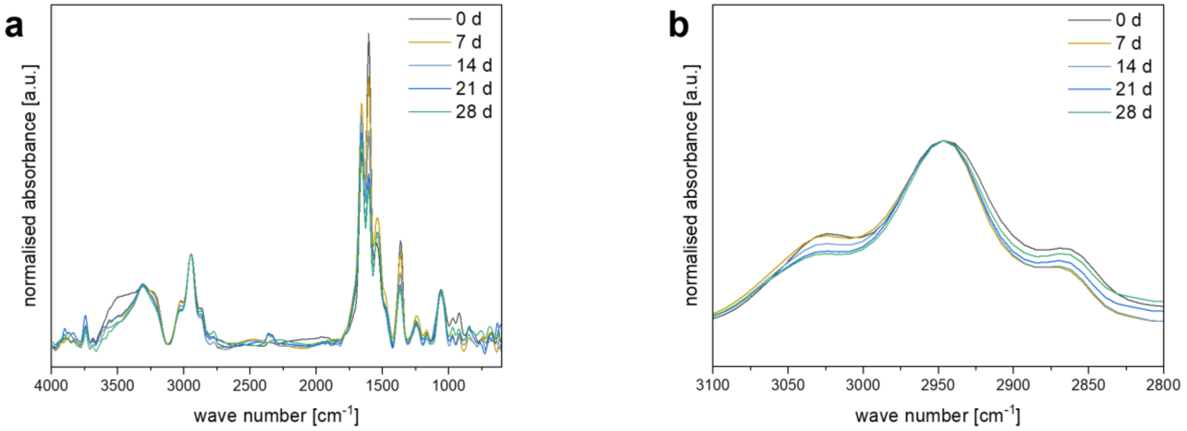

Figure S27 Stability study via FTIR spectra of PNCB2-co-BP networks at pH 7.4 over 28 days. The full spectra (a) and the zoom into the C-H stretching region (b).

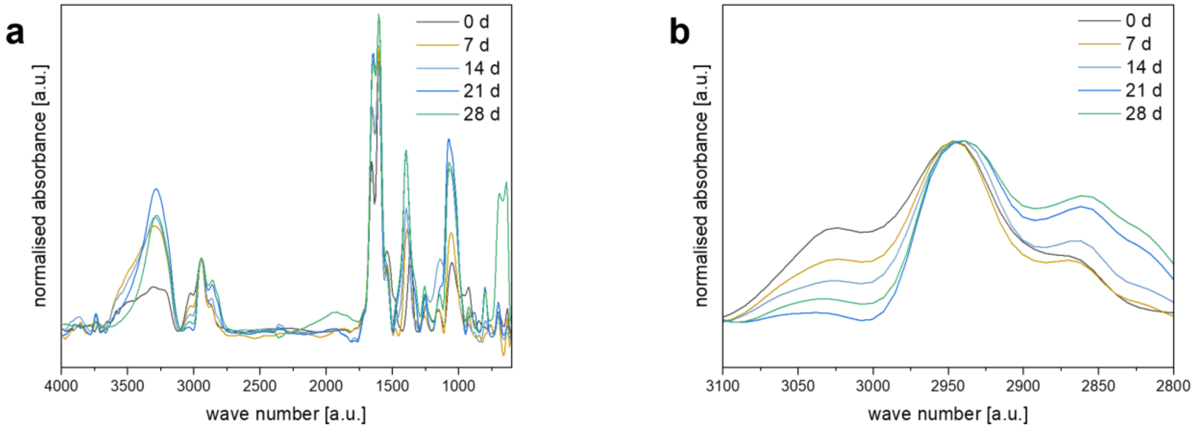

Figure S28 Stability study via FTIR spectra of PNCB2-co-BP networks at pH 8 over 28 days. The full spectra (a) and the zoom into the C-H stretching region (b).
